# Evidence-based scientific thinking and decision-making in everyday life

**DOI:** 10.1186/s41235-024-00578-2

**Published:** 2024-08-07

**Authors:** Caitlin Dawson, Hanna Julku, Milla Pihlajamäki, Johanna K. Kaakinen, Jonathan W. Schooler, Jaana Simola

**Affiliations:** 1 Department of Education, University of Helsinki, Siltavuorenpenger 3A, 00170 Helsinki, Finland; 2 Department of Psychology and Speech-Language Pathology, University of Turku, 20014 Turku, Finland; 3 INVEST Research Flagship Center, University of Turku, 20014 Turku, Finland; 4 Department of Psychological and Brain Sciences, University of California Santa Barbara, Building 251, Santa Barbara, 93106 USA

**Keywords:** Decision-making, Epistemic cognition, Individual differences, Motivated reasoning, Thinking styles, Curiosity, Scientific evidence, Information-seeking

## Abstract

In today’s knowledge economy, it is critical to make decisions based on high-quality evidence. Science-related decision-making is thought to rely on a complex interplay of reasoning skills, cognitive styles, attitudes, and motivations toward information. By investigating the relationship between individual differences and behaviors related to evidence-based decision-making, our aim was to better understand how adults engage with scientific information in everyday life. First, we used a data-driven exploratory approach to identify four latent factors in a large set of measures related to cognitive skills and epistemic attitudes. The resulting structure suggests that key factors include curiosity and positive attitudes toward science, prosociality, cognitive skills, and openmindedness to new information. Second, we investigated whether these factors predicted behavior in a naturalistic decision-making task. In the task, participants were introduced to a real science-related petition and were asked to read six online articles related to the petition, which varied in scientific quality, while deciding how to vote. We demonstrate that curiosity and positive science attitudes, cognitive flexibility, prosociality and emotional states, were related to engaging with information and discernment of evidence reliability. We further found that that social authority is a powerful cue for source credibility, even above the actual quality and relevance of the sources. Our results highlight that individual motivating factors toward information engagement, like curiosity, and social factors such as social authority are important drivers of how adults judge the credibility of everyday sources of scientific information.

## Significance statement

A sharp rise in misinformation, especially online, has made it increasingly difficult to find and use trustworthy information. Recent work suggests that the ways people engage with scientific evidence to make decisions is driven by individual differences in beliefs and attitudes about knowledge, thinking styles, ideologies, and personality. However, much of the previous decision-making work has focused on critical thinking interventions in educational settings or economic decision-making tasks.

We address the need for more naturalistic research around information-related decisions by examining how adults investigate scientific evidence from real news articles in order to make a voting decision on the issue of peat renewability, which has had significant political, environmental and economic impacts in Finland.

With a combination of data-driven and theory-driven analytical approaches, we examine a wide range of individual differences which have been implicated in scientific thinking and decision-making, and we also maintain rigorous methodological standards by testing pre-registered hypotheses. We demonstrate that curiosity and positive science attitudes, cognitive control, prosociality and emotional states, were related to engaging with information and discernment of evidence reliability, more than numerical skills. We disentangle the concepts of scientific and social authority and show that people are more likely to believe that information from sources that have social power, like well-known news outlets, is more credible. Finally, we show that subject knowledge and demographic factors are critical drivers of people’s decisions on science-related issues and highlight the unclear relationships between information engagement, evidence evaluation, and real decision outcomes.

## Introduction

The ability to understand and critically evaluate scientific information impacts political campaigns, legislature, public health, and everyday decisions that affect one’s personal wellbeing. Increased accessibility of information means that everyone has greater exposure to varying qualities of information, underlining the importance of the ability to discern good quality scientific evidence and evaluate scientific argumentation, especially when making meaningful decisions such as choosing to take a vaccine or to vote for environmental policy measures (Cacciatore, 2021; Lewandowsky et al., 2012). Public discourse on science and information literacy has increased in response to an increase in misinformation and fake news, and the importance of being able to identify credible information sources has stoked interest in ways to enhance numeracy and cognitive reasoning skills to better equip people to interpret scientific information.

However, recent work suggests that how people seek and use information may also be affected by individual differences in personality traits, thinking styles and dispositions, and even beliefs and ideologies (Appelt et al., 2011; Bruine de Bruin et al., 2020; Choung et al., 2020). These differences impact how people engage with scientific evidence to support their existing knowledge and form new concepts (Kiili et al., 2008). They can influence whether people tend to trust their own intuitions or seek validation from experts, whether they enjoy mulling over complex problems or prefer simple solutions, and whether they are willing to change their mind when encountering evidence that contradicts their prior beliefs.

In this study, we used a combination of data-driven and hypothesis-driven methods to investigate how individual differences in cognitive skills, thinking styles, traits, and beliefs affect how people evaluate the credibility of scientific information and then use that information to make a real science-related decision. We conceptualized a large set of individual difference variables as a psychological space where the potentially overlapping and interrelated constructs represent dimensions relevant to scientific information seeking and decision-making (Eisenberg et al., 2019). We then tested how these dimensions might predict specific behaviors in the decision-making process, such as time spent seeking information and evaluations of source reliability.

### Scientific evidence evaluation

The theoretical framework behind modern science literacy currently lacks consensus, but most scholars agree that it goes beyond the idea of possessing knowledge to encompassing how people interact with scientific information, the methods by which scientific information is produced and shared, the agents and goals driving those processes and their motivations and biases, and how information is used in different modalities such as print press versus digital medias (Sharon & Baram-Tsabari, 2020; Sinatra & Lombardi, 2020; Sinatra et al., 2014). Importantly, it emphasizes that an understanding of how scientific information interacts with the structures and processes of civic society has consequences for how we navigate a modern world where our important decisions are driven by engagement with science and technology (Howell & Brossard, 2021).

Evaluating scientific argumentation, apart from other types of persuasive argumentation, requires contextualizing claims within the specific methodologies of science (Faize et al., 2017; Sutter, 2006). High-quality scientific evidence requires specific constraints such as controlled research environments, replicable results, and claims that are supported by a consensus of qualified experts. Features of the information source like author expertise and affiliations or document type can be especially important in determining evidence quality where readers have less prior content knowledge or where there is conflicting information available (Hämäläinen et al., 2020). Studies in adolescents show that a majority of students struggle to accurately gauge the expertise and reliability of a source and the impact of the author’s perspective on the way information is presented (Coiro et al., 2015). Students tend to focus on the content and accept insufficient evidence for author expertise, such as claims made by the authors themselves, the fact that the information appeared on a public website, the quantity of the information, or even the idea that the new information just “makes sense” (Coiro et al., 2015). Evidence evaluation is not only a problem for students; laypeople (in contrast to professional fact checkers and domain experts) rarely corroborate evidence from multiple sources, and individuals can differ greatly in their evidence evaluation strategies (Kammerer et al., 2021).

Understanding scientific evidence also requires a tolerance for complexity, uncertainty, disagreement, and theory updating (Howell & Brossard, 2021; Sinatra et al., 2014). A common misconception is that lack of total certainty in a theory implies a lack of scientific consensus, or even that the claims are insubstantiated or untrue (Sinatra et al., 2014). People who believe that scientific knowledge should be absolutely certain truth are less likely to be persuaded by scientific messaging communicating uncertainty and less likely to seek more information, and often find contradictory scientific evidence frustrating and untrustworthy (Bråten et al., 2011; Rabinovich & Morton, 2012). This low confidence in scientific evidence often stems from a misunderstanding of the scientific method, which does not aim to make truth claims but uses probability-based statistical procedures to test the likelihood of particular effects in a controlled environment. The intrinsic uncertainty in scientific evidence can make misinformation more appealing when it is presented as simple and certain.

These skills as a matter of public information literacy can have significant effects on societal decision-making, as the appearance of expertise can be used to manipulate public opinion; for example, industry lobbies can make scientific claims which may be understood as credible simply because of their investment in a topic rather than because of real field expertise or credentials (Sinatra & Lombardi, 2020). Misunderstanding the goals and particularly the limitations of scientific claims can lead to mistrust in scientific institutions and less engagement with credible scientific information.

### Individual differences in information engagement

The ability to use scientific evidence in decision-making is partly driven by individual differences in beliefs and attitudes about science and, more broadly, about knowledge, which have been shown to predict voting behaviors, vaccine hesitancy, susceptibility to fake news, and epistemically suspect beliefs like paranormal beliefs and conspiracy beliefs (Lindeman et al., 2023; Rizeq et al., 2021). These differences affect how and why people seek information and how they interact with information sharing channels.

The driving force to seek information is curiosity, which is characterized as a motivational state toward exploratory behavior driven by intrinsic or extrinsic goals, and involves a feeling of reward with new knowledge acquisition (Murayama, 2022; Murayama et al., 2019). Curiosity is considered to be an important driver for learning as it is associated with enhanced memory for information and better learning outcomes (Gruber & Ranganath, 2019; Gruber et al., 2014; Kang et al., 2009; Mead et al., 2018; Metcalfe et al., 2020).

In our work, curiosity is relevant to how and why people are motivated to seek new knowledge, a type of curiosity known as epistemic curiosity. Epistemic curiosity is a framework to understand curiosity specifically toward knowledge, as opposed to other sensory experiences (Litman & Spielberger, 2003). In this work, epistemic curiosity is broadly conceptualized as a motivation toward knowledge and information engagement as both a trait which can be measured as an individual difference variable and a dynamic state which may fluctuate during information processing and between different contexts. Epistemic curiosity is often subdivided into two facets: interest curiosity and deprivation curiosity (Litman, 2008). Interest curiosity represents the internally motivated drive to acquire information purely for the joy of learning. It tends to be broad in topic, rather than focused, and is associated with positive emotions, mastery-oriented learning, analytical thinking, and enjoyment of problem-solving and deep thought (Cacioppo & Petty, 1982; Litman, 2008; Litman & Mussel, 2013). Deprivation curiosity, in contrast, represents a focused search for a particular answer and tends to be associated with negative emotions such as anxiety and discomfort with uncertainty, and a success-oriented mindset for learning (Litman, 2008). Deprivation curiosity prompts people to find answers in order to end the uncomfortable state of uncertainty brought on by a knowledge gap.

Uncertainty is an inherent part of the scientific method, as experimental effects can only be estimated, theories are constantly being updated, and many phenomena are not fully understood. When seeking information, people vary in their feelings of discomfort around this kind of uncertainty, known as need for closure. Need for closure motivates people to end the information-seeking process, sometimes by settling on the nearest acceptable answer regardless of its quality, which may in part account for why deprivation curiosity has also been associated with a failure to adequately scrutinize fake news(Roets & Van Hiel, 2011; Zedelius et al., 2021). Need for closure is associated with a tendency to be persuaded by superficial cues in persuasive argumentation rather than argument strength (Klein & Webster, 2000). When evaluating evidence quality, epistemic curiosity drives a person to engage with information and need for closure motivates them toward ending the engagement by finding a suitable answer.

A major issue with information engagement is that people tend to seek out and remember claims that confirm their prior beliefs. Seeking new information involves an element of open-mindedness to be aware of a gap in knowledge and a willingness to accept new information. Openness to experience has been operationalized as a personality trait that represents a predilection toward new experiences and ideas (Gosling et al., 2003), but that openness may not come with scrutiny or good judgment of the quality of those ideas. An actively open-minded thinking (AOT) style involves expressly seeking out and evaluating new information, especially information that might conflict with prior beliefs; in other words, it encapsulates the ability to reason analytically and resist bias when integrating new information (Svedholm-Häkkinen & Lindeman, 2018). It is associated with fewer anti-science attitudes, conspiracy beliefs and paranormal beliefs, and lower susceptibility to fake news (Pennycook & Rand, 2019; Rizeq et al., 2021). People high in actively open-minded thinking are more likely to seek out evidence against their prior beliefs and consider it fairly, keeping an open mind to new ideas.

However, being faced with a new epistemic situation or having one’s beliefs contradicted can feel challenging, bringing up negative emotions like defensiveness and even feeling threatening to one’s identity (Leary, 2018). Being able to make use of new information first requires a realistic perspective on one’s own current state of knowledge. The concept of intellectual humility was taken from the idea of general humility as an accurate view of oneself in relation to others, and applied to the relationship a person has with their own knowledge (Krumrei-Mancuso & Rouse, 2016). Intellectually humble people are aware of their own epistemic fallibility, have respect for others’ opinions and do not find new or contradicting information threatening, which may motivate them to search for and integrate new ideas and spend more time objectively evaluating source quality (Braasch, 2023). High intellectual humility has been associated with more open-minded and analytic thinking, intellectual engagement, curiosity, and higher scrutiny in evaluating scientific information (Huynh & Senger, 2021; Koetke et al., 2022; Krumrei-Mancuso et al., 2020; Porter & Schumann, 2018). We are interested in how these characteristics related to thinking styles and epistemic emotions might contribute to how people evaluate information, especially controversial science-related information which can activate core beliefs and ideologies about oneself and one’s social group.

### Numeracy

Evaluating the accuracy of scientific evidence requires understanding of mathematical concepts, which often involve statistical calculation of likelihoods, generalizations from samples to populations, and decisions which try to maximize benefits while minimizing risk. People use these concepts to understand everyday issues like their risk for health conditions, their role in environmental concerns, and how population-level political decisions will affect them.

Basic mathematical education provides a foundation for these skills, but cognitive biases can disrupt the reasoning process. Biases are the systematic errors made when applying heuristics, which are mental shortcuts that promote fast, intuitive, adaptive reasoning. Heuristic thinking steers decisions toward options that are more intuitive, easier, faster, more attractive, more familiar, more popular, and that reinforce a person’s perception of themself that they are a person who makes good decisions (Ceschi et al., 2019). They are particularly salient under pressured conditions like lack of time or lack of subject knowledge and affect how people interpret decision spaces (Bago et al., 2020). Education is also not enough to resist biased reasoning: some mathematical biases have been shown to actually increase after statistics education (Morsanyi et al., 2009).

Importantly, they do not simply indicate incorrect mathematical calculations; rather, biased reasoning in understanding scientific evidence usually represents a systematic misunderstanding of probabilistic events, disrupting one’s ability to use one’s mathematical skills to accurately gauge probabilities and risks (Morsanyi et al., 2009). An example of such a case would be a person deciding not to take a vaccine against a contagious deadly disease because their friend had a serious but very rare side effect from a vaccine, a situation which activates a number of cognitive biases. A personal experience or a powerful story about a rare negative outcome can make the possibility of further negative outcomes seem more likely because it is salient in memory (availability heuristic), is likely to get more attention in media and thus seem more common than it is (false consensus and base rate neglect), and seem riskier because it involves taking an unfamiliar action rather than doing nothing or taking a known risk (omission bias and ambiguity aversion) (Azarpanah et al., 2021). Numeracy as the ability to apply a mathematical way of thinking to a wide range of real situations and resist biased thinking is therefore a core component of science literacy and is critical for understanding scientific information.

### Rationality, reasoning, and thinking styles

Modern decision research makes a clear distinction between the idea of intelligence and the idea of rationality. While measures of intelligence are often correlated with performance on reasoning tasks, rationality is the idea that a decision maker can accurately weigh the utility and probability of possible outcomes according to one’s own goals (Stanovich, 2016). Stanovich (2016) discusses the cognitive scientific approach to two types of rationality, epistemic rationality and instrumental rationality, in terms of “what is true” and “what to do.” In other words, in order to make rational decisions, we must first understand the reality of the situation and then choose the option that best suits our values and goals. In the vaccine example given previously, the overall population risk of serious adverse consequences from the disease is much greater than the risk of adverse events from the vaccine. If one’s goal is to avoid serious illness, then a rational decision is likely to take a vaccine despite the low risk of a serious reaction. However, if one has a medical condition that is known to make adverse reactions to vaccines more likely, they might choose to avoid the vaccine and cope with the risk of disease using other strategies. In either case, a rational decision first requires an accurate appraisal of risk probability, which often involves more cognitive effort to overcome misinformation that exploits cognitive biases.

Theories of rational thought have dealt extensively with the idea of a dual-process model in which there are two systems, types, or modes of thought: one representing a fast, intuitive, heuristic style and one representing a slow, analytical, normative style that requires more cognitive effort. Reflectiveness, the recognition of the need to re-evaluate automatic or intuitive responses, is sometimes separated out into a third distinct function (Erceg et al., 2022; Pennycook et al., 2015; Stanovich, 2011).

The debate continues as to whether these models are necessary or sufficient to explain human reasoning (Evans & Stanovich, 2013). However, many thinking behaviors do not fit well into dual-process models that align analytical thinking with rationality along a single dimension with analytical thinking directly opposed to intuitive thinking. Rationalization and motivated reasoning, for example, are thinking styles in which reflectiveness allows a decision-maker to exploit reasoning skills toward justifying their existing behaviors and beliefs, disregarding conflicting evidence (Kahan et al., 2013; Newton et al., 2023). Some studies suggest that analytical thinking can actually lead to greater polarization on controversial topics, as individuals are motivated to apply their sophisticated reasoning skills toward maintaining ideologically congruent beliefs. By seeking out only information that supports their claims and rationalizing contradicting information, they accumulate evidence that supports positions important to their social and political ingroups, with the end result that intuitive and analytical thinking both lead to maintaining the same prior beliefs (Kahan & Corbin, 2016; Kahan et al., 2012, 2017; Sinatra et al., 2014). Moreover, while intuitive thinking (in contrast to analytical thinking) has been associated with irrationality and less effective decision-making, it is not always inaccurate. In natural decision-making situations, domain experts often accumulate enough subject knowledge that their intuitive judgments can efficiently solve problems in their field, again showing that a strict dichotomy between analytical and intuitive thinking styles is less useful in real-life contexts (Klein, 2008).

Other studies suggest that analytical thinking styles, and actively open-minded thinking in particular, may protect against motivated reasoning. Stenhouse et al. (2018) point out that actively open-minded thinking involves an additional element of explicitly seeking out and fairly considering evidence that contradicts prior beliefs, leading to less bias and greater resistance to misinformation. It has been linked to science endorsement (Pennycook et al., 2020) and fewer conspiracy beliefs (Pennycook et al., 2020; Stanovich & Toplak, 2019), as well as belief in human-caused climate change independently of media use, scientific knowledge or political ideology (Stenhouse et al., 2018).

A central question is whether individuals do not or cannot activate effective thinking styles when evaluating scientific evidence. Some studies suggest that failure to analyze information results mainly from limitations in an individual’s analytical skills. Martire et al. (2020) found that endorsers and non-endorsers of implausible claims were both more convinced by high-quality evidence than low-quality evidence, showing that people who endorse implausible claims are able to detect differences in evidence quality but not able to use analytical skills as effectively. Others suggest that one’s activation of analytical thinking is situational and can be influenced by context. Directing people’s attention to content accuracy, for example, can improve the quality of information they choose to share online, as they are primed to put more weight on the information quality (Pennycook & Rand, 2022, 2021; Pennycook et al., 2021).

These kinds of phenomena have led to multidimensional models of thinking styles that redirect the focus from the dichotomy of intuitiveness versus rationality to more clearly meaningful constructs such as open-mindedness and preferences toward effortful thinking (Newton et al., 2023). In the vaccine example, thinking styles that are more reflective, analytical, and crucially, open-minded, help a person re-evaluate their quick initial responses, put in the effort to fact-check information, and make sure that their beliefs are consistent with observable reality and that their actions are consistent with their goals. It is likely that whether people are willing and able to apply this kind of thinking may depend both on their individual dispositions as well as contexts that may or may not motivate them to prioritize information quality (Huynh & Senger, 2021; Kahan, 2013; Koetke et al., 2022; Martire et al., 2020; Pennycook & Rand, 2019, 2022).

### Cognitive and perceptual processes

Insights into different reasoning processes important to evidence-based decision-making have also come from the field of cognitive neuroscience. When answering scientific questions, experts show more inhibition against strong initial intuitive (but naïve) responses than novices, in order to activate the correct mental model (Masson et al., 2014; Potvin et al., 2014). This suggests that mathematically and scientifically correct mental models do not replace naïve theories but exist alongside them; the ability to choose the correct model then depends on inhibiting irrelevant or incorrect information and directing attention to the correct information. Inhibition and updating have been implicated as executive functions important for critical thinking, and constructs like cognitive control, emotion regulation, and experience explain individual differences in decision-making competence better than intelligence (Bruine de Bruin et al., 2020; Erceg et al., 2022; Li et al., 2021).

The ability to flexibly direct attention, adapt one’s thinking to new rules, integrate new information, shift a pre-existing concept, or apply new knowledge is known as cognitive flexibility (Ionescu, 2012). Aspects of cognitive flexibility like divergent thinking help decision-makers find innovative and creative solutions to a problem. While convergent thinking focuses on finding well-defined and typical solutions, divergent thinking allows a decision maker to push the boundaries of the decision space and explore new ideas, and is associated with the ability to more flexibly control attentional focus (Ionescu, 2012; Zabelina & Ganis, 2018). Although it might seem counter-intuitive, flexibility can help decision-makers re-evaluate their prior beliefs and integrate new information for a better understanding of their options. This process of exploring new solutions can sometimes lead to a “aha moment” or experience of insight, which is highly rewarding. The experience of insight may come from perceptually driven cognitive conflict and restructuring of the mental representation when new information becomes available, leading to a different understanding of the context and the subjective feeling of insight (Danek & Flanagin, 2019; Tulver et al., 2023). However, the feeling of insight can be misleading and bias people toward believing that information accompanying a feeling of insight is always true, a phenomenon that can be exploited by sources of misinformation and disinformation (Laukkonen et al., 2019).

Interestingly, divergent thinking and insight-based problem solving are also associated with perceptual phenomena such as higher involuntary switching rates for bistable visual illusions, which appear to exist ambiguously between two different perspectives, such as a dancer who appears to spin both clockwise and counter-clockwise, and lower level pattern recognition such as discerning out-of-focus images (Blake & Palmisano, 2021; Laukkonen & Tangen, 2017; Schooler & Melcher, 1995).

Recent research suggests that even ideological profiles may be driven in part by these lower-level perceptual and cognitive processes through shared neural mechanisms for information processing (Zmigrod et al., 2021). One proposed theory, evidence accumulation, posits that the decision-maker samples from the decision space, gathering information until a threshold is reached and a decision can be made. Dynamics of these evidence accumulation processes have been shown not only in low-level perceptual decisions, but also value-based decisions and high-level dispositions and preferences such as risk-taking versus risk-avoidance, and can be measured through various methods including behavioral tasks and electroencephalography (Polanía et al., 2014; Usher et al., 2013; Zmigrod et al., 2021). Cognitive inflexibility on behavioral tasks, for example, predicts extremist ideologies such as willingness to die for one’s social ingroup, suggesting that there may be a connection between the rigidity of cognitive processes and ideological dogmatism (Zmigrod et al., 2019, 2019). Similarly, caution in speeded decision tasks is associated with political conservatism and higher need for certainty and security, potentially indicating a globally cautious approach strategy to decision situations, regardless of context (Zmigrod et al., 2021).

Understanding the impacts of brain functioning on very high-level tasks such as decision-making works toward answering the question of what cognitive resources people have available for reasoning. Some of these processes, such as cognitive flexibility, may be able to be improved with targeted interventions, while others shed light on reasoning phenomena such as biases that have their roots in the efforts of the human brain to conserve energy, helping us to understand why they are so resistant to change and adding a dimension to the explanation of why people make the decisions they do.

Together, the body of literature suggests that reasoning and decision-making are driven by a complex interaction of cognitive processes, topic knowledge and literacy, attitudes and beliefs, traits, and emotional states, especially on topics that are ideologically polarized or personally relevant, like climate change and health. Beliefs, attitudes and values about knowledge can impact which information sources people trust, how people share information, how satisfied they are with the knowledge they have, how likely they are to change their mind, and even which reasoning skills and styles they are likely to use (Lindeman et al., 2023). Understanding interactions of the many individual factors that drive people’s engagement with scientific information allows us to better understand the use and misuse of scientific evidence in everyday life.

### The present study

Evidence-based decision-making is at the heart of scientific literacy. While previous work has illuminated the interactions between some individual differences in information engagement and thinking styles (e.g., Braasch 2023; Lindeman et al. 2023; Pennycook and Rand 2019; Zedelius et al. 2021), and their effects on evidence evaluation, the present study aims to take a more comprehensive view by incorporating a large set of individual differences and using these variables to predict the same participants’ behavior on a realistic evidence evaluation and decision task.

Because there is not yet a unified theoretical framework that incorporates all of the constructs of interest, we began with a data-driven approach in order to structure this very complex conceptual space. Each variable represents a unique component of a cognitive style or behavior related to decision-making, and we chose versions of each task or questionnaire carefully to avoid redundancy in the constructs under investigation. However, we also considered it important to include a broad set of variables with potentially overlapping constructs in order to represent the nuances of what is likely a multidimensional space (Newton et al., 2023). We predicted that relationships between these variables and decision-making behavior would be driven by higher-order latent factors which comprise combinations of highly correlated individual variables.

To investigate these questions, we pre-registered this study in two parts. In the first part, we investigate whether there is a latent structure in the set of variables previously implicated in information engagement, through exploratory factor analysis (https://osf.io/g2mpe). This latent variable method reduces a high-dimensional space with many related and overlapping constructs into a less complex structure in order to clarify the impact of influential individual differences on evidence evaluation and decision-making.

In the second part, we examined the predictive power of these factors on behavior in a realistic decision-making task in which participants read news articles about an environmental topic that varied in quality and were then asked to make a voting decision based on that information (https://osf.io/dtqax). We examined whether people are generally able to discern the quality of scientific information sources and whether individual differences in cognitive skills and dispositions can predict behaviors on the task, such as time spent engaging with different qualities of evidence. We predicted that individual differences related to positive beliefs and attitudes toward science and information, such as epistemic curiosity and actively open-minded thinking, would predict more time engaging with information and more accurate discernment of evidence quality.

## Methods

### Preregistration

This study was preregistered in two parts: https://osf.io/g2mpe and https://osf.io/dtqax. All materials, data, and code are available from the Open Science Framework repository (https://osf.io/72mut/).

### Participants

Participants were recruited through university student mailing lists, university researchers and teachers, social media channels, and word of mouth. The study was positively reviewed by the University of Helsinki Ethical Review Board in Humanities and Social and Behavioural Sciences.

Out of the initial set of 177 participants, 174 participants completed at least part of the study with valid responses. Three participants’ datasets were not valid, either because the participants self-reported so in the validity check (*n*=2, “Ignore my material. Some other reason prevented me from participating properly” and “Ignore my material. For the most part, I didn’t concentrate or read the questions properly.”) or because they exited the study before completing the first questionnaire (*n*=1). All participants indicated their Finnish language level as native or conversational. Age ranged from 19 to 74 years old (*M* = 49, *SD* = 16.1). Of the valid datasets, 119 participants identified as women, 41 as men, three as non-binary, three as another gender not listed, and eight preferred not to say. Education level was skewed toward higher education, likely due to the sampling method; 16 participants reported their highest level of education as doctoral or licentiate degree, 75 participants had a master’s degree, 38 had a bachelor’s degree, 10 had a vocational school degree, 27 had completed a matriculation examination, four had primary or elementary school education, one chose “none of the above/no formal degree,” and three chose “other education.” Two of the participants who chose “other education” gave additional information in the text box: a few years of college-level study, and an older Finnish title describing education between vocational and university degrees.

In our pre-registration, we identified a sample size of around 170 participants as sufficient for factor analysis with the full set of 34 variables. Justifications for the ideal sample size for exploratory factor analysis are inconsistent, based on the strength of the data (Costello & Osborne, 2005), data missingness or analysis methods (McNeish, 2017). The rule-of-thumb suggests item/subject ratios of at least 3–5 times the number of variables. We also considered the following: 1. Moderate to high expected communality among variables, as many of our variables have been shown to be correlated in previous research; 2. Overdetermination of factors: we expected a few major factors would emerge from our large set of variables, with many variables loaded onto each factor, again because of theoretical predictions about the relationships between our variables; 3. Careful treatment of missing data, maximum likelihood estimation, oblique factor rotations.

### Materials

We designed an online study comprised of a series of questionnaires, cognitive behavioral tasks, and the citizen’s initiative decision-making task based on a real public petition. All materials were presented in Finnish. Data were collected through the online platform Gorilla from September 2021 through February 2022. The order of the tasks and questionnaires was pseudo-randomized so that participants always completed the demographic information and citizen’s initiative task first, and a dataset was only considered if the participant had completed the citizen’s initiative task. Participants were allowed to complete the tasks and questionnaires at their own pace and could complete the study in about 90 min. However, they were encouraged to take breaks, and therefore some participants chose to complete the different tasks and questionnaires over a few hours in their own time. Full materials in Finnish and English are available at the Open Science Framework repository https://osf.io/72mut/.

#### Citizen’s initiative task

The citizen’s initiative task uses a real issue taken from a Finnish petition website maintained by the Finnish Ministry of Justice https://www.kansalaisaloite.fi/fi/aloite/7914. In spring 2021, two opposing environmental petitions were published: one in favor of declaring peat as a renewable resource and one against declaring peat as a renewable resource. We chose this issue as a relevant real-world voting decision, expecting that most people in Finland would be somewhat aware of the issue but it would be a less polarizing topic than, e.g., vaccines. During the task, participants read six real articles from news and information websites and online science magazines. The sources and their categories were identified by consensus of three independent raters using slightly modified criteria from Sutter (2006) and the CRAAP method (Blakeslee, 2004). The sources were divided into two types: authority sources, which were authored by people and institutions with power, such as Amnesty International, and personal sources, which were authored by individuals as opinions or personal interest stories. Within each type, the sources varied in quality: credible sources were balanced and well-researched; non-credible sources were biased or badly referenced; and irrelevant sources were about the topic of peat but not about peat renewability. The presentation order of the 6 articles was randomized between participants. Partway through data collection, one of the articles was taken down from its hosting site, so we reproduced it as a PDF and some participants read it fixed in last position (*n*=94).

During the task, we first gave a brief neutrally worded description of peat and the petition in support of declaring peat as a renewable resource. Then, participants were presented sequentially with links to the six articles. After reading each source, participants were asked to evaluate the source on its convincingness, expertise, reliability, and shareability on 5-point Likert scales. After the initial introduction of the task and after each source, participants were also asked to rate their familiarity, interest, and curiosity in the topic of peat as a natural resource on 5-point Likert scales, the likelihood that they would support the petition on a 5-point Likert scale, and their emotional arousal and valence on a 9-point visual Self-Assessment Manikin (SAM) (Lang et al., 2008). Participants’ reading times were recorded for each article as the time between the click to open the link and the click to progress to the next page, then divided by the number of words per source to obtain reading rates in words per minute (WPM) for each source per participant, which accounted for the different lengths of the articles.

#### Individual difference variables

Individual differences consisted of numeracy operationalized as understanding probability-related mathematical concepts, trait variables such as fluid intelligence and thinking styles, and cognitive and perceptual phenomena such as cognitive flexibility, which are operationalized through measurements taken from cognitive behavioral tasks (Table 1).

#### Numeracy

 We measured understanding the concepts of randomness and probability score with a count of total correct responses on 4 selected items measuring understanding of the mathematical concepts of randomness and probability (Fiedler et al., 2017).

The heuristic reasoning task included 6 items testing the tendency to give biased answers for probability related mathematical questions due to heuristic thinking (Morsanyi et al., 2009). The response options are normative (mathematically correct), incorrect, or heuristic. Three of the items prompt the equiprobability heuristic, and three items prompt the representativeness heuristic. The heuristic reasoning scores were converted to binary variables due to the high skew of responses (mostly normative). A score of 1 reflects at least one heuristic response for each category, equiprobability (H-E score) and representativeness (H-R score).

#### Individual trait variables

 Fluid intelligence was measured with Raven’s standard progressive matrices short form, comprised of graphics that illustrate a progressive series of logical relationships (Raven et al., 1992). Participants were asked to find the next graphic piece to continue the series. The short form has 9 items and is scored as a count of total correct responses (Bilker et al., 2012).

The following scales were self-reported Likert scales with five, six, or seven response options.

The comprehensive intellectual humility scale (IH) is comprised of 22 items which load onto 4 factors: independence of intellect and ego, openness to revising one’s viewpoint, respect for others’ viewpoints, and lack of intellectual overconfidence (Krumrei-Mancuso & Rouse, 2016). It is scored in four parts as a sum of items for each factor.

The actively-openminded thinking short scale (AOT) is comprised of 7 items reflecting an actively openminded thinking style and scored as a sum of responses (Haran et al., 2013).

The ten item personality index (TIPI) is comprised of two items for each of five factors: agreeableness, emotional stability, extroversion, openness, and conscientiousness (Gosling et al., 2003). It is scored as a mean of item responses per factor.

The need for cognition scale short form is comprised of 18 items reflecting enjoyment and preference for challenging cognitive activities, scored as a sum of item responses (Cacioppo et al., 1984).

The need for closure scale short form is comprised of 15 items reflecting discomfort with ambiguity and a preference for stable, predictable, unchallenging knowledge, scored as a sum of item responses (Roets & Van Hiel, 2011).

The epistemic curiosity scale short form (EC) is comprised of 10 items divided into two factors: interest curiosity and deprivation curiosity (Litman, 2008). It is scored as a sum of item responses per factor.

The science curiosity scale is comprised of 4 items modified from Landrum et al. (2016) and Motta et al. (2021) and is scored as a sum of item responses.

The science attitudes scale (SA) is comprised of 8 items modified from Archer et al. (2015) reflecting science identity and the importance of science in everyday life, and 4 items reflecting trust in science and scientists modified from Nadelson et al. (2014).

#### Perceptual and cognitive behavioral tasks

 In the Go/No Go task, participants are asked to accurately detect target (Go) stimuli while inhibiting responses to distractors (No Go stimuli). The letters T, H and N were presented on a screen for 250ms, and after another 750ms the screen proceeded to the next trial. Participants were asked to press a button if they saw T or H (Go stimuli) but to do nothing if they saw N (No Go stimulus). There were 400 randomized trials in total. Outcome variables are sensitivity (*d’*: *z*-value of hit rate minus false-alarm rate), as a general measure of target detection adjusted for overall response rate, and bias, which is a measure of how liberal or conservative a participant’s response patterns are overall; in other words, how likely they are to respond, regardless of whether the item is a target or distractor (Stanislaw & Todorov, 1999).

The Navon letters task provides a measure of one’s ability to focus either on a global or local perceptual level. In each randomized trial, participants were shown large alphabetical letters arranged from smaller letters. In the first block, they were instructed to identify the larger letters by pressing the appropriate key on a keyboard, and in the second block they were instructed to identify the smaller letters. Block order was randomized between participants. Each block had 60 trials and each stimulus was shown for 250ms. The global–local precedence index quantifies the bias toward the global processing level, where responses are typically faster to targets when they are presented as large letters than as smaller letters. The global-to-local interference index indicates the extent to which a global bias interferes with processing targets at the local level.

The unusual uses task (UUT) is a measure of divergent thinking that asks a person to propose as many possible uses for everyday items such as a brick or a newspaper (Guilford, 1967). Participants were asked to type their responses and were given two minutes per item: a shoe, a newspaper, a board, and a paperclip. The measures of interest are a count of valid responses (fluency) other than the original use, and the number of unique categories of responses (flexibility). Flexibility is scored according to functionality categories, e.g., using a newspaper as a hat and using it as a sock represent two uses of the same functional category (clothing) and would thus earn only one point.

The Necker cube appears as a two-dimensional wireframe drawing of a transparent three-dimensional cube that appears to spontaneously switch between two different subjective orientations (Necker, 1832). When viewing the Necker Cube, people tend to perceive the image as spontaneously switching back and forth between two different orientations (with the front side facing down to the left, or up to the right). Participants were asked to view the cube and press a button on a keyboard every time they experienced a switch.

**Table 1:** Operationalization of cognitive and perceptual task variables

Task	Variable	Description	Psychological construct
Go no-go task	Sensitivity (*d’*)	Z value of the hit-rate minus that of the false-alarm rate	General measure of target detection ability, adjusts for overall response rate; based on correctly identifying targets and correctly ignoring non-targets
	Bias ($\beta$)	Ratio of normal density functions at criterion of Z values used to calculate *d’*	Measure of how liberal or conservative response patterns are. Liberal observers are more likely to respond overall and have values approaching 0; conservative observers are less likely to respond and have values that increase over 1; unbiased values approach 1
Unusual uses test	Fluency score	Count of all valid responses	Measures spontaneous cognitive flexibility
	Flexibility score	Count of unique categories of given responses	Ability to generate ideas from different categories
Navon local-global processing task	Global-local precedence index	Standardized mean difference (Cohen’s *d*) in reaction time between global and local judgments on consistent trials only	Quantifies the bias toward a global processing level
	Global-to-local interference index	Standardized mean difference (Cohen’s *d*) in reaction time between inconsistent and consistent correct trials in local condition only	Positive values indicate the extent to which the bias toward global stimuli interferes with processing local information
Necker cube task	Perceptual switching rate	Switches per second calculated from two 30-second sessions of viewing the Necker Cube	Perceptual switching has associated with divergent thinking and insight-based problem solving

## Data cleaning and quality checks

### Data exclusions and missing data

Self-report questionnaires and tasks were processed separately. Given that most of the derived variables depend on sum or average scores, a participant’s data were excluded for an individual task or questionnaire if the measure was incomplete, indicating they exited the study during that measure. Data were also excluded measure-wise based on specific comments in the open response box at the end of the study, e.g., if a participant indicated that there was a technical issue in a specific measure, or the participant thought their data for that measure was invalid for some other reason. No participant data were excluded from the Citizen’s Initiative task based on responses.

Two participants responded that their education level was “none of the above/no formal degree” or “other, what?” with no additional information. Since these responses did not fit into any clear category, these participants were removed from the regression analyses.

Scale reliability was checked for all individual factors of scales using Cronbach’s alpha and McDonald’s omega, with the exception of the Ten Item Personality Index, which is not designed to optimize internal reliability but rather validity (Gosling et al., 2003). All scales had alpha values above 0.6, with the exception of the trust in science and scientists subscale (4 items), with $\alpha$ = 0.58 (Appendix 1).

Median completion times and response distributions were checked for unusual response behavior, and no data were removed based on these methods (see pre-registration https://osf.io/g2mpe). Participant data were removed from the matrix reasoning task if median reaction time was 500 ms or lower. Data were excluded by trial from Go/No Go and Navon if reaction time was 150ms or lower or if the reaction time was outside total trial length indicating technical error. We excluded a participant’s entire dataset from Go/No Go and Navon if responses had less than 60% accuracy (percentage of correct trials) and from Navon if the same response was given on 95% or more trials. 3.08% of data were excluded from Go/No Go and 1.33% from Navon.

Invalid responses were removed for the Unusual Uses Test during scoring (e.g., nonsense). Variables with inter-measure correlations $>.85$ were removed arbitrarily: UUT fluency was removed due to high correlation with UUT flexibility (*r* =.98). The final full set of variables and their correlation matrix is described in Appendix 2.

Participant datasets were excluded from further analysis if they had a high proportion of missing and low-quality data in either of the questionnaire or task sets (questionnaires $>32\%$ missing, *n*=21; tasks $>30\%$ missing, *n*=12). Missing data were examined for patterns related to demographics. Of participants with at least one missing variable, only the number of missing task variables was correlated positively with age (*r*(21) =.49, *p* = 0.017).

### Data transformations

Due to very uneven numbers of participants at each education level, the levels were recoded into three categories: Upper secondary school or lower (including vocational school) (n=42), university degree (n=40), or graduate degree (master’s and doctorates/licentiates) (n=92).

The following variables were log-transformed due to absolute skew $>1$: Go/No Go bias, Navon global to local interference, Navon global to local precedence, and Necker cube switching rate.

## Statistical analysis

We first reduced the dimensionality of the psychological space with exploratory factor analysis, which uses correlations between sets of items to find the latent structure that best explains their groupings. For scales with an internal structure, individual factors of the scale were modeled separately, e.g., the five personality traits from the Ten Item Personality Index were included as separate variables. This is because factors within existing scales are thought to capture distinct constructs and might have unique relationships with other variables. Because the variable set includes continuous and binary variables, which complicates the correlation matrix and factor score calculations, we used functions from the lavaan package typically used in confirmatory factor analysis to fit the exploratory factor analysis model (Rosseel, 2012). Factor scores were extracted using the Empirical Bayes Modal method using the lavPredict function in lavaan.

Linear mixed effects models were fitted using the lme4 package (v1.1.34; Bates et al. (2014)) in R statistical analysis software (v4.1.3; R Core Team (2021)). These models were used to test our hypotheses about the individual differences that predict evidence evaluation ratings and reading rates. We used the lmerTest package to apply Satterthwaite’s method for estimating degrees of freedom and generate p-values (Kuznetsova et al., 2017). The afex package was used to generate F values for significance tests of model terms (Singmann et al., 2023). Post hoc pairwise contrasts were performed using estimated marginal means with the emmeans package (Lenth, 2016). Likert scores were treated as continuous. Multicollinearity was checked for all models, which showed low or moderate variance inflation factor, VIF < 10 (Lüdecke et al., 2021).

Each model is formulated according to a specific hypothesis in our preregistered analysis plan https://osf.io/dtqax. Some adjustments were deemed necessary and the following changes were made after the preregistration. For reading rates which are positively skewed, we fit generalized linear mixed effects models with a gamma distribution and log link function and with age as a scaled continuous variable. It should be noted that while very short reading times are valid for our research questions (choosing to not read a source at all is an extreme version of not reading carefully), the longer reading times could indicate either spending a long time on the source (valid) or taking a break (invalid). Since it was not possible to identify clear outliers and the reading times were skewed, we did not attempt to remove outliers. Instead, the model distribution takes them into account. For the evidence accumulation models, the pre-registered plan to re-code the sources by a ranked quality order was somewhat arbitrary. Instead of this two-way interaction of ranked quality by time, we used a three way interaction between time, source type, and source quality. Finally, as a means of simplifying the model structure and ensuring the inclusion of all participants, we chose to exclude gender as a predictor from all models. This decision was influenced by the uneven distribution of gender categories and the intention to avoid the exclusion of any categories with few participants. Notably, we initially tested the ratings and reading time models with the two largest gender categories (women/men) and found no effect of gender.

In the results section, model summaries are shown with authority as the reference level for source type, credible as the reference level for source quality, and upper secondary school (or lower) as the reference level for education.

## Results

In the interest of space, we report here the pre-registered results that showed effects, and in a later section we report additional exploratory analyses. For the full set of planned analyses including those which showed null results, see the pre-registration (https://osf.io/dtqax).

### Creating a psychological space: Should task and questionnaire variables be combined in factor analysis?

The first question was whether our cognitive behavioral tasks and self-report questionnaires measured constructs within the same psychological space. The structuring of a psychological space involves quantifying the relationships between the initial variables and combining them based on extraction of latent constructs that reflect those relationships (Eisenberg et al., 2019).

Theoretically, if cognitive strategies reflected in perceptual and behavioral responses might be related to thinking styles, ideologies, beliefs, and personality traits, then they may be able to be combined into one psychological space and analyzed together. However, the measurement methods and interpretations of task and questionnaire variables are very different in practice. Cognitive behavioral task performance in this study reflects reaction times and accuracy of fast, automated processes like inhibition and target discrimination, while the questionnaires are self-reported preferences, attitudes and beliefs on Likert scales, requiring intentional introspection, or sum scores on tests of mathematical reasoning or logic such as Raven’s matrices. Following Eisenberg et al. (2019) and Zmigrod et al. (2021), we used a few different approaches to determining whether task and questionnaire measures were related enough to justify considering them within a single psychological space.

The correlation matrix (excluding the binary variables H-E and H-R) showed generally lower correlations between variables of different measure type than the same measure type, indicating weaker relationships between task and questionnaire variables than within type (see Appendix 2).

Additionally, we used 10-fold cross-validated ridge regression to obtain $R^2$ for each of four predictive categories: task-by-task, questionnaire-by-questionnaire, task-by-questionnaire, and questionnaire-by-task, leaving out the two binary variables (H-E and H-R). With this method, each variable is held out in turn and used to predict the other variables in the same category (tasks or questionnaires) and the opposite category. Variables showed weak predictive ability between-measure when compared to within-measure (Table 2).

The lack of correlation and weak predictive ability between measurement types indicates that the task and questionnaire variables should be analyzed separately.

**Table 2:** Predictive ability within and between measurement types

Direction	Mean $R^2$	SD $R^2$
Questionnaire by questionnaire	0.34	0.15
Questionnaire by task	0.042	0.054
Task by task	0.53	0.31
Task by questionnaire	0.092	0.084

### Exploratory factor analysis

The main hypothesis for the exploratory factor analysis was that there are latent structures that drive relationships between the many related constructs we are interested in. Based on previous literature, we predicted that a generally science-positive attitude would be related to understanding randomness and probability, high intellectual humility, need for cognition, open-minded thinking, epistemic curiosity, and cognitive flexibility, a science-negative attitude toward science would be related to lower openmindedness and intellectual humility, greater deprivation curiosity and need for closure, and less cognitive flexibility. The resulting factors were later used in linear mixed effects models to predict behavior on the citizen’s initiative task.

Because the task variable set was comprised of derived variables from only four tasks with high correlations between variables from the same task, we conducted EFA only on the 22 questionnaire variables. Since EFA requires a complete dataset, participants with any missing variables were removed for this analysis, resulting in (*n*=127).

Bartlett’s test of sphericity and the Kaiser–Meyer–Olkin test indicated that the questionnaire set might be factorable (mean measure of sampling adequacy for questionnaires = 0.63), keeping in mind that these tests are less reliable for larger sample sizes. Parallel analysis, Very Simple Structure, and Empirical Bayes Information Criterion indicated 5, 3, or 4 factors, respectively. While we stated plans to evaluate model fit in the preregistration, recent work suggests that these fit indices are inappropriate for exploratory factor analysis (Montoya & Edwards, 2021). Therefore, we used the previously mentioned measures and theoretical coherence to decide on the number of factors.

**Table 3:** Factor loadings for the four-factor structure

Variable	Prosociality	Curiosity	Openmindedness	Cognitive Skills
Respect for others’ viewpoints (IH)	**0.59**	0.10	− 0.03	0.02
Agreeableness (TIPI)	**0.40**	− 0.26	− 0.06	0.07
Openness (TIPI)	**0.40**	0.31	0.32	− 0.22
Need for closure	**− 0.38**	− 0.22	− 0.10	0.08
Extraversion (TIPI)	**0.30**	0.16	− 0.11	− 0.15
Science identity (SA)	− 0.15	**0.81**	− 0.22	0.10
Need for cognition	0.20	**0.77**	0.1	− 0.04
Interest curiosity (EC)	0.26	** 0.64**	0.28	0.07
Science curiosity	− 0.12	**0.60**	0.09	0.04
Deprivation curiosity (EC)	0.02	**0.50**	0.24	− 0.05
Importance of science (SA)	− 0.24	**0.43**	0.21	− 0.01
Independence of intellect and ego (IH)	0.15	** 0.35**	− 0.12	− 0.03
Trust in science (SA)	− 0.27	**0.30**	0.31	− 0.02
Heuristic-equiprobability	− 0.16	**− 0.19**	0.13	− 0.18
Openness to Revising One’s Viewpoint (IH)	0.11	0.10	**0.58**	0.14
Conscientiousness (TIPI)	0.11	0.23	**− 0.53**	0.00
Actively openminded thinking	− 0.11	0.12	** 0.46**	0.20
Emotional stability (TIPI)	0.31	0.09	**− 0.41**	0.12
Lack of intellectual overconfidence (IH)	0.09	− 0.34	**0.36**	0.17
Randomness-probability	− 0.20	0.18	0.10	**0.70**
Heuristic-representativeness	0.27	− 0.12	− 0.03	** 0.66**
Matrix reasoning	0.00	0.04	0.09	** 0.42**

Multidimensional scales are shown with their original factors separately: Intellectual Humility (IH), Ten Item Personality Index (TIPI), Science Attitudes (SA), Epistemic Curiosity (EC). Loadings in bold font show factor assignments

The most coherent structure was a four-factor solution (Table 3). In exploratory factor analysis, items are sometimes removed if they load below a certain threshold or if they load highly on two or more factors (cross-load) (Costello & Osborne, 2005). Because we were not creating a new scale from these factors but rather using the factor scores in other analyses on the same sample, we considered it best to not remove any items. This also follows the approach by Eisenberg et al. (2019) and Zmigrod et al. (2021) who used a large battery of cognitive tests and questionnaires and did not remove items based on loadings. Since participant factor scores act as weights in our further analysis, the contribution of low-loading items to the results is minimal and the contribution of cross-loading items is better representative of reality than if they were removed in order to create more conservative factors.

#### Factor 1: prosociality

The first factor is comprised of traits mainly associated with prosocial behavior: respect for others’ viewpoints from the comprehensive intellectual humility scale, agreeableness, openness and extraversion from the ten item personality index, and need for closure. Need for closure loads negatively onto this factor, indicating greater tolerance for uncertainty. Interest curiosity, representativeness heuristic and emotional stability cross-load positively on this factor; however, importance of science and trust in science cross-load negatively. A high score for this factor suggests an interest in and tolerance of other people’s opinions and experiences.

#### Factor 2: curiosity

The second factor is driven by multiple curiosity measures and measures of engagement with science and knowledge: both interest and deprivation components of epistemic curiosity, science curiosity, all three dimensions of the science attitudes scale (science identity, importance of science and trust in science), need for cognition, and independence of intellect and ego from the Comprehensive Intellectual Humility Scale, and heuristic-equiprobability (negative). Lack of intellectual overconfidence and need for closure cross-load negatively, suggesting a confidence, possibly even overconfidence, in one’s intellect and some tolerance for uncertainty. Agreeableness cross-loads negatively, while openness cross-loads positively on this factor. The negative loading for the equiprobability heuristic indicates fewer heuristic errors. Altogether, this factor suggests positive self-image associated with science, high general curiosity, and a confident positive attitude toward science and learning.

#### Factor 3: openmindedness

The fourth factor includes actively open-minded thinking as well as openness to revising one’s viewpoint and lack of intellectual overconfidence from the intellectual humility scale. While the personality trait of openness to experience cross-loads positively on this factor, suggesting some overlap in constructs, the intellectual humility items alongside the actively open-minded thinking scale indicate that this factor captures a particular aspect of intellectual open-mindedness, distinct from general openness, that reflects a willingness to change one’s mind in the face of new information. Two personality items also load negatively on this factor: conscientiousness and emotional stability. In some studies, conscientiousness has been associated with more conservative ideologies or response styles and extreme conscientiousness may approach dogmatism or obsessiveness (Carter et al., 2016). Low conscientiousness may therefore represent a more flexible and less dogmatic viewpoint. The negative loading of emotional stability items from the TIPI suggest that intellectual openmindedness has an aspect of emotional arousal.

#### Factor 4: cognitive skills

The third factor includes items related to cognition and reasoning: fluid intelligence as measured by the matrix reasoning task, understanding of randomness and probability, and heuristic reasoning (heuristic-representativeness and cross-loading with heuristic-equiprobability). The positive loading for the randomness-probability and matrix reasoning scores indicates that more normative responses on these questions are associated with higher fluid intelligence. However, the heuristic reasoning scores (H–R and H-E) are binary scores with 1 representing at least one heuristic error; therefore, the positive loading for H-R indicates a likelihood of making at least one heuristic error. This might seem counter-intuitive, but the response distributions for these questions was such that most people 59.09% for H-R and 61.04% for H-E) make at least one error, so a possible explanation is that cognitive skills like fluid intelligence and explicit understanding of probabilities are not closely related better resistance to heuristic reasoning. This is in accordance with prior research showing that resistance to heuristic reasoning is specifically associated with critical thinking skills, apart from general intelligence and education level, and is persistent even after training (Morsanyi et al., 2009). The equiprobability heuristic is particularly resistant to change, and as H-E loads poorly and negatively; this may indicate that it has less in common with the other reasoning items and partially explain its cross-loading. Notable other loadings were openness (negative) and actively open-minded thinking (positive).

### Source evaluation in the citizen’s initiative task

The main aim of the citizen’s initiative task was to determine whether people could discriminate between sources of different levels of social authority and different content quality. The models described here were pre-registered in the second phase of the study (https://osf.io/dtqax).

We predicted that in general, people would rate authority sources (versus personal) and credible sources (versus non-credible and irrelevant) higher in convincingness, expertise, and reliability, indicating an ability to discern more expert and better quality evidence from poor quality evidence.

Three linear mixed effects models were fit with source convincingness ratings, expertise ratings, and reliability ratings as dependent variables and with source type and source quality and their two-way interaction as fixed effects. Age and education level were additional fixed effects in all models, and a random intercept was included at the participant level in all models (model summary tables found in Appendix 3).

**Fig. 1:**
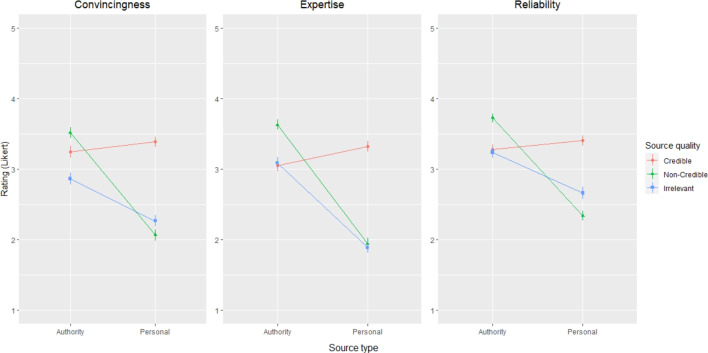
Interactions of source type and source quality for ratings of convincingness, expertise and reliability. Error bars indicate standard error of the mean

Results for convincingness, expertise and reliability showed similar patterns (Fig. 1). For all three ratings, there was an interaction between source type and source quality: convincingness (*F*(2,849) = 64.45, *p* $<0.001$), expertise (*F*(2,849) = 111.1, *p* $<0.001$), and reliability (*F*(2,822) = 70.03, *p* $<0.001$). For the authority sources, the non-credible source was consistently rated the highest, followed by the credible and irrelevant sources. In contrast, for the personal sources, the credible source was rated the highest followed by the non-credible and irrelevant sources. In general, participants were better able to distinguish the credible information when the source was personal rather than from an authority. We computed post hoc comparisons using estimated marginal means, with differences in most pairwise combinations, which can be found in Appendix 4.

Participants with higher education gave lower convincingness (*F*(2,167) = 3.21, *p* = 0.043) and reliability ratings (*F*(2,169) = 6.68, *p* = 0.0016). Specifically, those with a graduate degree gave lower convincingness ratings (b = $-$0.24, 95%CI = [$-$0.42, $-$0.05], SE = 0.09, *t* = $-$2.53, *p* = 0.012), while those with a lower university degree gave lower expertise ratings (b = $-$0.25, 95%CI = [$-$0.48, $-$0.01], SE = 0.12, *t* = $-$2.09, *p* = 0.037).

Older participants gave higher expertise (b = 0.0075, 95%CI = [0.00, 0.01], SE = 0.0026, *t* = 2.89, *p* = 0.0045) and reliability ratings (b = 0.0056, 95%CI = [0.00, 0.01], SE = 0.0023, *t* = 2.46, *p* = 0.014).

As the texts differed in length, we also considered the potential effect of text length on ratings. A comprehensive examination of the text length effect is available in Appendix 5.

### Reading rates in the citizen’s initiative task

We hypothesized that if participants recognized that authority sources and credible sources contained better quality information relevant to the task, then they would spend more time carefully reading these sources compared to the other source categories, i.e., they would have slower reading rates.

A generalized linear mixed effects model was fit with reading rates (in words per minute) as a dependent variable and with source type and source quality and their two-way interaction as fixed effects. Age and education level were additional fixed effects, and a random intercept was included at the participant level (model summary tables found in Appendix 3).

Reading rates were predicted by source authority ($\chi ^2$(1,N=171) = 79.53 *p* $<.001$) and quality ($\chi ^2$(2,N=171) = 106.96, *p* $<0.001$). Participants spent more time reading authority sources. Pairwise contrasts for source quality showed differences between all pairs. The non-credible sources were read at the fastest rate, followed by the credible sources and then the irrelevant sources (Table 4). Text length clearly had an effect as well, with more time spent on longer sources (Table 5). Age did not have an effect, but graduate school education level predicted slower reading rates (b = $-$0.45, SE = 0.20, *t* = $-$2.26, *p* = 0.024).

**Table 4:** Pairwise contrasts between levels of source quality for reading rates (WPM), averaged over the levels of the source type and education

Contrast	Estimate	SE	df	z ratio	*p*
Credible–non-credible	− 0.24	0.067	Inf	− 3.65	0.0008***
Credible–irrelevant	0.46	0.066	Inf	7.032	$<.001$***
Non-credible–irrelevant	0.70	0.067	Inf	10.50	$<.001$***

P value adjustment: Tukey method for 3 tests. Tests are performed on the log scale. ***Indicates *p* < 0.001

**Table 5:** Raw reading times/rates per source in minutes (top) and words per minute (bottom)

Source type	Source quality	Num. words	Mean (SD)	Median	Min	Max
*Reading times in minutes*						
Authority	Credible	376	3.93 (22.38)	1.68	0.03	291.24
Authority	Non-credible	1146	5.77 (6.36)	5.18	0.04	73.96
Authority	Irrelevant	327	3.83 (8.33)	2.44	0.03	78.25
Personal	Credible	892	5.61 (14.79)	3.32	0.03	154.54
Personal	Non-credible	422	2.0 (3.29)	1.12	0.02	31.14
Personal	Irrelevant	408	2.3 (3.3)	1.8	0.03	37.62
*Reading rates in words per minute (WPM)*						
Authority	Credible	376	609.75 (1393.83)	224.28	1.29	12287.58
Authority	Non-credible	1146	995.09 (3329.83)	221.36	15.49	27860.62
Authority	Irrelevant	327	485.92 (1297.79)	133.88	4.18	9790.42
Personal	Credible	892	1535.40 (4245.92)	268.37	5.77	26583.22
Personal	Non-credible	422	1296.16 (2486.36)	377.21	13.55	17858.65
Personal	Irrelevant	408	592.92 (1525.09)	226.37	10.84	14097.32

Reading time was recorded as the time between clicking on the link and clicking the button to progress to the next page

### Effects of individual differences in information-seeking styles and cognitive skills on ratings and reading rates

An important question was whether individual differences identified by our task measures and factor analysis would be associated with patterns of behavior in the citizen’s initiative task. If thinking styles, dispositions, attitudes, and cognitive skills affect how people engage with and process scientific information, then we would expect to find a difference in outcomes on this task.

Of the four identified factors, we did not find effects for the openmindedness and cognitive skills factors, nor for other task variables included in the planned analyses; therefore, those models are not reported here. The models described here, as well as those that did not show any results, were pre-registered in the second phase of the study. All planned analyses are described in the pre-registration (https://osf.io/dtqax). Model summary tables can be found in Appendix 3.

#### Curiosity factor

We predicted that higher curiosity factor scores would predict higher ratings of source expertise and reliability for the credible sources, more careful reading, higher ratings for topic interest and topic curiosity, and more positive emotional valence, indicating that people high in curiosity and positive science attitudes would be more attuned to quality information, spend more time information-seeking, and feel more positive about the process.

Five linear mixed effects models were fit with ratings for source expertise, source reliability, topic interest, topic curiosity, and emotional valence as dependent variables. An additional generalized linear mixed model was fit with source reading rates (in words per minute) as the dependent variable. Fixed effects were curiosity factor scores, source type, and source quality and their three-way interaction. Age and education level were additional fixed effects in all models, and a random intercept was included at the participant level in all models (model summary tables can be found in Appendix 3).

**Fig. 2:**
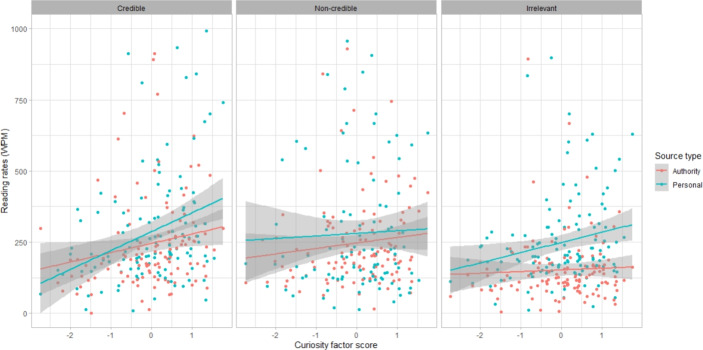
Interactions between source type and source quality, and source quality and curiosity factor scores for reading rates. Shaded area indicates confidence intervals. Outlying data points have been cropped (*n*=60 or 8%)

Higher curiosity factor scores predicted higher reading rates for the credible sources and the personal irrelevant source (Fig. 2), with interactions between source quality and curiosity factor scores at the non-credible quality level ($\chi ^2$(16,N=171) = 15.12 *p* = 0.00052), and source type and source quality at the personal and non-credible levels ($\chi ^2$(16,N=171) = 8.23 *p* = 0.016).

**Fig. 3:**
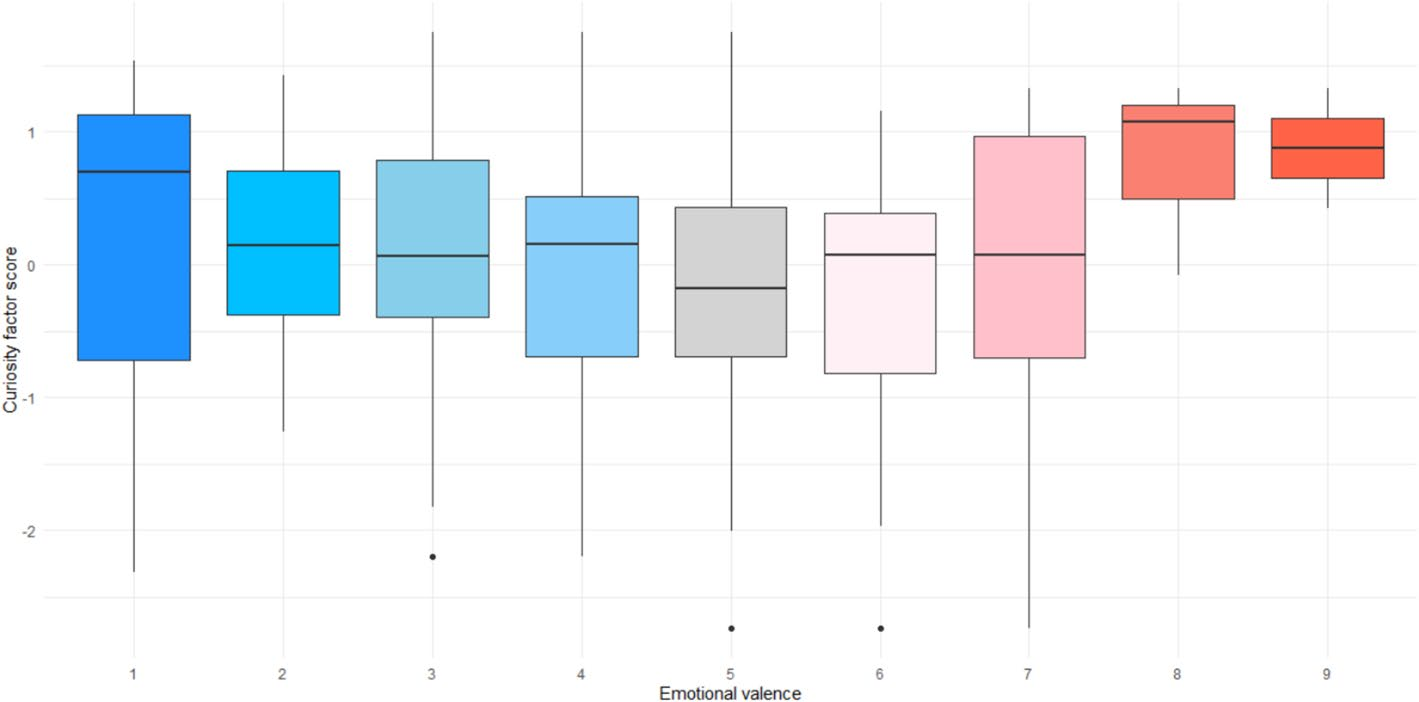
Nonlinear relationship of curiosity factor scores and emotional valence ratings, rated on a 9-point Self-Assessment Manikin with endpoints Unpleasant (1) to Pleasant (9)

**Fig. 4:**
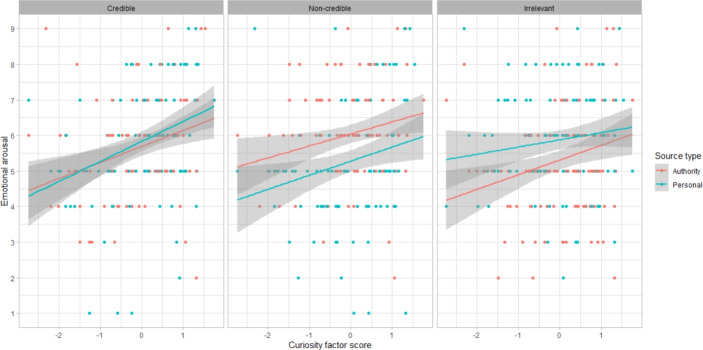
Higher curiosity factor scores predicted higher emotional arousal ratings, rated on a 9-point Self-Assessment Manikin with endpoints Peaceful (1) to Agitated (9). Shaded area indicates confidence intervals

There was an effect of curiosity factor scores on emotional valence (b = $-$0.34, 95%CI = [$-$0.66, $-$0.02], SE = 0.16, *t* = $-$2.1, *p* = 0.037). However, it appears that the relationship may not be linear: high curiosity factor scores were associated with both the most unpleasant and the most pleasant emotional states (Fig. 3). Indeed, an additional (non-preregistered) exploratory model using the same fixed effects with emotional arousal as the dependent variable showed that participants with higher curiosity factor scores rated their emotional arousal as higher (b = 0.47, 95%CI = [0.16, 0.77], SE = 0.15, *t* = 3.01, *p* = 0.0029) (Fig. 4).

#### Prosociality factor

We hypothesized that higher prosociality factor scores would predict higher ratings for ratings of convincingness and shareability and higher emotional arousal, since people higher in the prosociality factor might be more attuned to the social elements of engaging with and sharing information.

Three linear mixed effects models were fit with source convincingness, source shareability, and emotional arousal as dependent variables. Fixed effects were prosociality factor scores, source type, source quality, and their three-way interaction. Age and education level were additional fixed effects in all models, and a random intercept was included at the participant level in all models.

Higher emotional arousal was predicted by prosociality (b = 0.43, 95%CI = [0.1, 0.76], SE = 0.17, *t* = 2.59, *p* = 0.01) and age (b = 0.019, 95%CI = [0.00, 0.03], SE = 0.0074, *t* = 2.49, *p* = 0.014). People higher in prosocial personality traits and older people reported higher emotional arousal during the task.

Contrary to our hypotheses, convincingness and shareability were not predicted by prosociality factor scores.

Interestingly, shareability ratings were instead predicted by education (*F*(2,123) = 5.18, *p* = 0.0069) as well as the interaction of source type and quality (*F*(2,630) = 30.09, *p* < 0.001). People with less education were more likely to say they would share the sources. The credible sources were rated more shareable than the other categories with the exception of the authority irrelevant source, which was also rated as shareable.

#### Cognitive control

We hypothesized that the ability to correctly respond to targets and inhibit responses to false alarms in a Go/No Go task would be associated with more careful reading and more accurate evidence evaluation, i.e., greater sensitivity in a response inhibition task would predict higher ratings of source expertise and source reliability, and slower reading rates.

Three linear mixed effects models were fit with source expertise, source reliability, and source reading rates as dependent variables. Fixed effects were sensitivity (*d’*), source type, and source quality and their three-way interaction. Age and education level were additional fixed effects in all models, and a random intercept was included at the participant level in all models.

**Fig. 5:**
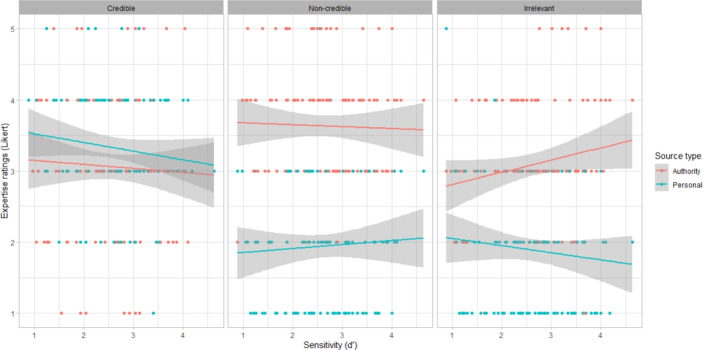
Interactions of sensitivity with source quality for expertise ratings

Source expertise ratings were predicted by an interaction between sensitivity and source quality for the irrelevant sources (b = 0.23, 95%CI = [0.00, 0.46], SE = 0.12, *t* = 1.97, *p* = 0.049). Higher sensitivity predicted higher expertise ratings for the authority irrelevant source and lower ratings for the personal irrelevant source (Fig. 5).

**Fig. 6:**
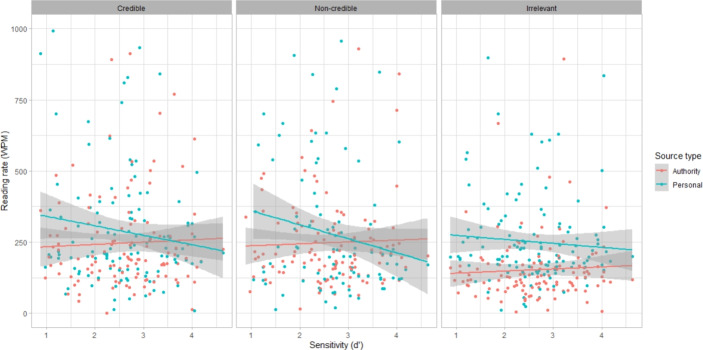
Interactions of sensitivity with source type and source quality for reading rates. Shaded area indicates confidence intervals. Outlying data points have been cropped (*n*=60 or 8%)

For reading rates, there were interactions between sensitivity (*d’*) and source type ($\chi ^2$(1,N=126) = 7.86 *p* = 0.005) and source quality ($\chi ^2$(1,N=126) = 8.42 *p* = 0.015). People with higher sensitivity read the credible and non-credible personal sources more carefully (Fig. 6).

Contrary to our hypotheses, higher sensitivity did not predict reliability ratings.

#### Evidence accumulation

We were also interested in how people’s familiarity, interest, and curiosity on the topic of peat renewability might change over time as they gathered information from the articles they were reading. We hypothesized that all three topic-related ratings would increase over time during the task, and that differences in these ratings at each timepoint would be associated with more informative sources, i.e., ratings would be higher for a particular timepoint when given after authority versus personal sources, and after credible versus non-credible or irrelevant sources. This would indicate that people feel that they obtain more information on a topic from better quality and more expert sources.

Three linear mixed effects models were fit with topic familiarity, topic curiosity, and topic interest as dependent variables, with fixed effects of time point (1–6), source type, and source quality and their three-way interaction as fixed effects. Age and education level were additional fixed effects in all models, and a random intercept was included at the participant level in all models. Participant ratings were collected at each time point immediately after reading an article.

For topic familiarity ratings, there was an effect of time (*F*(5,821) = 2.23, *p* = 0.05). However, the effect was small as 60.81% of participants did not show a change in familiarity ratings between the first and final time points.

**Fig. 7:**
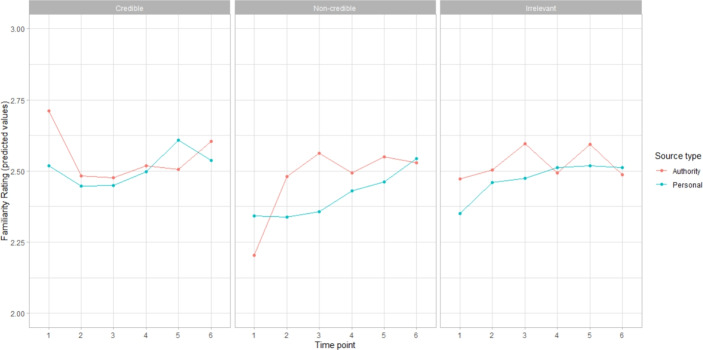
Model predicted values of topic familiarity over time by source categories. Note that y-axis has been adjusted to highlight effect

The plot shows a slight U-shaped curve for familiarity of the credible sources and an increase in familiarity over time for the personal non-credible and irrelevant sources (Fig. 7). There was also an effect of age (*F*(1,167) = 10.2, *p* = 0.0017), indicating that older participants rated the topic as more familiar than younger participants. There were effects of source type (*F*(1,821) = 4.59, *p* = 0.032) and source quality (*F*(2,821) = 4.57, *p* = 0.011). Familiarity ratings were higher after reading authority than personal sources, and higher after reading credible than non-credible and irrelevant sources, indicating that participants felt more familiar with the topic after reading higher-quality articles (Fig. 7). However, all effects were quite small.

There was no effect of time for topic curiosity or topic interest.

#### Exploratory analysis: support for reclassifying peat as renewable predicted by factors related to topic, sources, and personal background

While the primary analysis aimed to identify predictors of source evaluation and information seeking, we had no prior hypotheses about whether these behaviors were related to the decision outcome.

The final decision of whether or not to vote for the petition to classify peat as a renewable resource was measured with a 5-point Likert scale indicating support for the petition, where 1 represents less support for the petition (i.e., a pro-science viewpoint) and 5 represents more support for the petition (i.e., an anti-science viewpoint). We chose to measure petition support this way to capture an element of uncertainty rather than using a binary yes/no answer.

We collected petition support ratings at the beginning of the task and after each source for a total of 7 ratings. In order to determine whether petition support ratings showed an overall change over the course of the task, we subtracted the first rating from the final rating. Most participants’ ratings did not change at all from the beginning to the end of the task (68.46%). A smaller proportion either decreased their support rating by one point (18.79%) or increased by one point (7.38%). And finally, only a few participants decreased their support by two points (3.36%) or increased by two points (2.013%). The median response was 2 throughout the task. Therefore, we did not further investigate any effect of change in support over the task.

We fit a linear mixed effects model with petition support as the dependent variable and all source and topic related ratings (source convincingness, expertise, reliability, and shareability; topic familiarity, curiosity, and interest), emotional state ratings, the four factor scores, sensitivity (*d’*), and demographics (age and education level) as fixed effects and a random intercept was included at the participant level.

People who gave higher ratings for source shareability, topic interest and topic curiosity were more likely to support the petition more (shareability: *F*(1,652) = 4.05, *p* = 0.045; topic interest: *F*(1,7012) = 17.14, *p* $<0.001$; topic curiosity: *F*(1,743) = 8.06, *p* = 0.0047), while people with higher topic familiarity and education gave less petition support (topic familiarity: *F*(1,703) = 19.85, *p* $<0.001$; education: *F*(2,109) = 4.1, *p* = 0.019).

## Discussion

This study aimed to understand how individual differences in thinking styles and dispositions, attitudes toward information, and cognitive skills might be associated with information-seeking behaviors and evidence evaluation. We identify social authority as a key component of source features in evidence evaluation and highlight the importance of curiosity and emotional engagement in scientific decision-making.

### Evidence evaluation

**Fig. 8:**
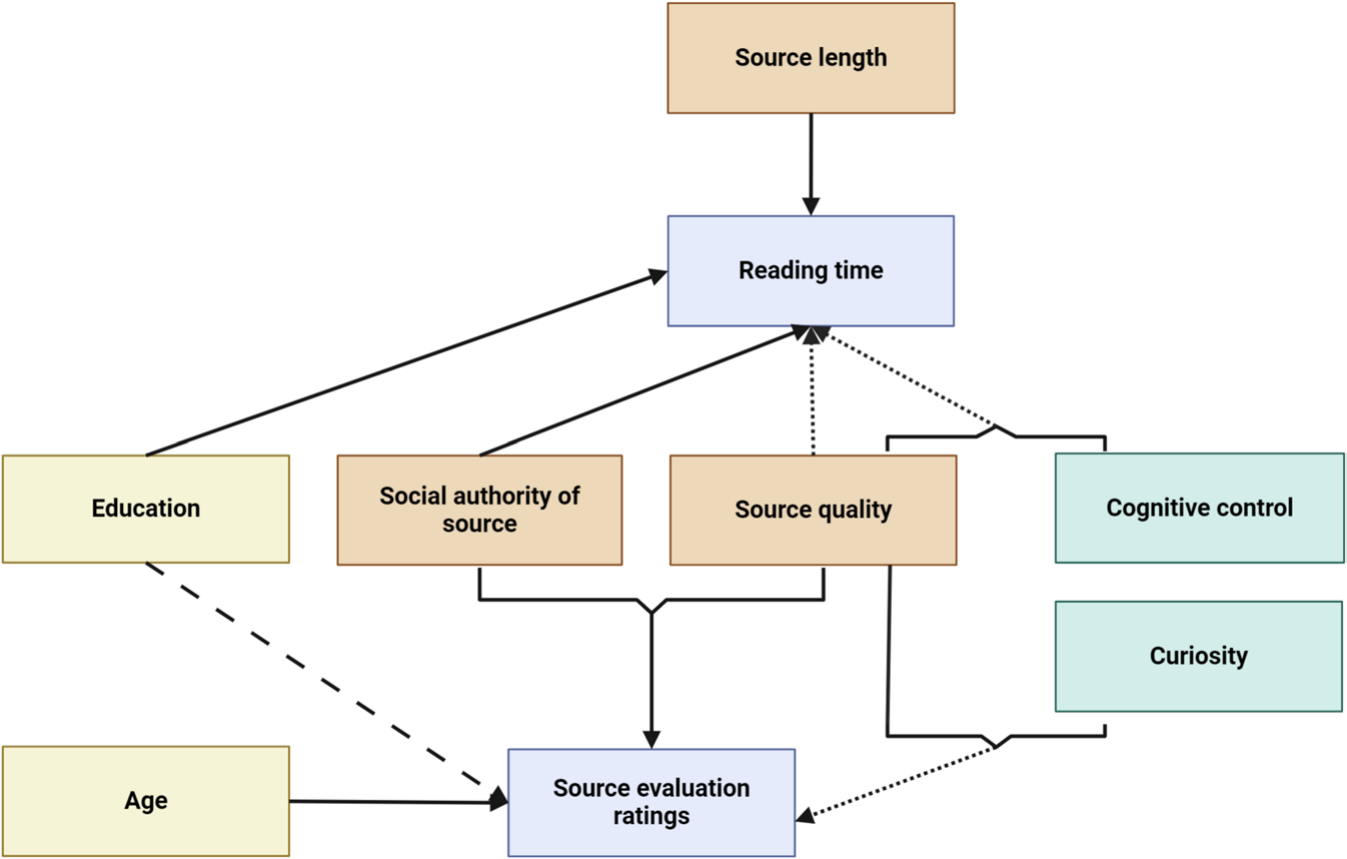
Conceptual diagram showing predictors of reading times and source evaluation ratings during the citizen’s initiative task. Solid lines represent positive effects; dashed lines represent negative effects; dotted lines represent relationships where the effect direction depends on the level of the variable. Brackets represent interactions. Colors indicate variable types: yellow are demographic variables; red are source features; teal are individual difference variables; blue are source evaluation behaviors used as dependent variables. Created with Biorender.com

Participants were generally able to rate better quality evidence higher in convincingness, expertise and reliability (Fig. 8). The similar pattern of results for the three ratings suggests that people evaluate source quality as a multidimensional construct; a good quality source has persuasive argumentation (i.e., it is convincing), the author has expertise, and the information is plausible or agrees with the reader’s prior knowledge (it is perceived to be reliable). These categories are similar to multidimensional models of epistemic justifications; for example, plausibility relates to personal justification, whereby a reader relies on their own opinions or knowledge as a justification for knowing something (Kammerer et al., 2021; Greene et al., 2008). Importantly, these justifications and dimensions of source quality are subjective and not necessarily correct, often due to the reader’s lack of domain expertise.

In particular, we were interested in the aspect of justification by authority, whereby the alleged expertise of the source serves as a proxy for evaluation of the content, especially when the evaluator’s own domain knowledge is insufficient or when they are unable to determine the information credibility from its content alone (Kammerer et al., 2021; Lucassen & Schraagen, 2013). Although our participants could clearly identify the credible personal source and rate it higher than the non-credible and irrelevant personal sources, they rated all three authority sources highly, suggesting that there is likely an important effect of authority.

Prior research has shown that students who use scientific authority as a justification give better quality source evaluations (Hämäläinen et al., 2021). Using source authority as a cue for quality can be an effective heuristic for quick decision-making in science, where credentials are often a trustworthy cue for reliability. But there is a growing problem of scientific and medical misinformation spread by the exploitation of professional credentials or scientific-sounding language to cue legitimacy while lacking the appropriate domain expertise (Di Domenico et al., 2022; Zaboski & Therriault, 2020). In this study, we disentangled an overall aspect of social power from the quality of the source content and showed that people are readily convinced by authority even when the content was unreliable and irrelevant to the task.

Our results concerning reading rates were more complicated than expected. Participants spent more time reading the articles that were from authorities, suggesting that people used social authority as a cue to read more carefully. This may help them decide whether information that initially appears plausible is, in fact, credible.

More time and effort spent on reading has been linked to better quality justifications, better multiple-text comprehension, and a greater tendency to value using multiple information sources, suggesting that critical and analytical evidence evaluation requires enough time spent engaging with information, particularly multiple sources (Bråten et al., 2014; Hämäläinen et al., 2021; Kammerer et al., 2021; Kiili et al., 2022). Shifting between contradicting pieces of evidence when gathering information is a key behavior for critical reading (Tsai et al., 2022). However, in many everyday situations, scientific information is gathered sequentially rather than in parallel, requiring different processes of integration and working memory to compare claims. In our study, cognitive control predicted more time reading better quality personal sources and higher expertise ratings for the authority irrelevant source only, which could indicate that participants with greater cognitive control pay more attention in more complex situations, as it could help them distinguish the conflicting cues of author expertise and content quality in the sources. In a saturated information environment, the ability to shift attention toward useful information and away from irrelevant, distracting, and misleading information may be an important skill for critical reading (Kozyreva et al., 2023). However, because cognitive control did not directly predict reliability ratings in our study, it is unclear what benefit this greater cognitive control might actually give to evidence evaluation here.

Participants with higher education levels were more likely to give overall lower ratings for convincingness, reliability, and expertise, suggesting that higher education is related to greater scrutiny of the content quality. In contrast, older participants were more likely to give higher expertise and reliability ratings overall. Education and age were positively correlated in our dataset, but the correlation was driven by graduate degrees: people with graduate degrees were more likely to be older than people with other education levels. We found that older people with graduate degrees demonstrated more scrutiny in their evaluations, while older people with lower education levels demonstrated less scrutiny.

The demographic effects likely reflect a lower level of media literacy in older adults with lower education, as prior research has found that older people are more likely to believe and share fake news and are less able to evaluate the credibility of online information sources (Guess et al., 2019; Rasi et al., 2019). Rasi et al. (2019) note that there is a troubling lack of comprehensive research on media literacy amongst older citizens. Older people need to be able to access media and communication technologies to maintain social networks, access personal health related information, and obtain reliable information to participate in a society where they play important roles in social and political decision-making. With increasing new technologies and a growing aging population, development of media literacy pedagogies targeted at older adults is a critical goal to address age-related inequalities (Rasi et al., 2019).

We show here that it is necessary to take into consideration the prior viewpoints, cognitive styles, beliefs, dispositions, and motivations such as curiosity and cognitive control, which can influence how people approach information that differs from their own knowledge base (Braasch, 2023). These results underline the need for rigorous study designs which isolate content and source features in order to identify their unique contributions to source evaluation and which include individual differences that may influence how people interact with information. We also corroborate previous work implicating source authority, time spent information-seeking, education level, and age as critical nodes for potential interventions among the adult population.

### Epistemic curiosity is associated with prosocial personality traits, emotional responses and information seeking behavior

Previous work has found that increased emotionality generally predicts belief in fake news; however, the specific emotions of interested, alert, determined, and attentive were not related to discernment of fake or real news (Martel et al., 2020). Interestingly, these positive emotional states are associated with epistemic curiosity. People who espouse epistemic beliefs such as that knowledge is simple and unchanging are more likely to experience negative emotions such as frustration and confusion when encountering complex or contradictory information, whereas curiosity is generally seen as a positive epistemic emotion and is associated with critical thinking and self-regulated learning (Chevrier et al., 2019).

**Fig. 9:**
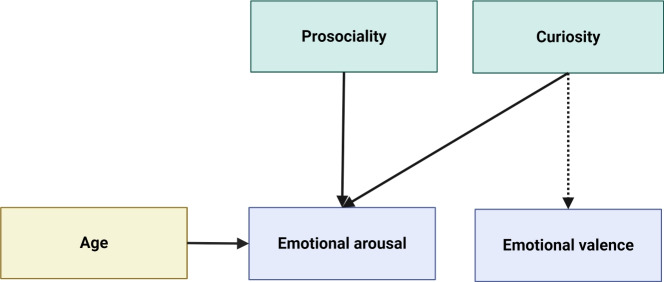
Conceptual diagram showing predictors of emotional states during the citizen’s initiative task. Solid lines represent positive effects; dotted lines represent relationships where the effect direction depends on the level of the variable. Colors indicate variable types: yellow are demographic variables; teal are individual difference variables; blue are emotional state ratings used as dependent variables. Created with Biorender.com

In this study, curiosity factor scores were associated with higher emotional arousal during the citizen’s initiative task, which likely indicates activation of epistemic emotions during the task due to the controversial and complex nature of the topic (Fig. 9). Higher prosociality factor scores also predicted higher emotional arousal. High emotional arousal can be associated with emotional dysregulation, which is related to low intellectual humility and reflects defensiveness and reliance on cognitive biases to dig further into a prior belief or stance in order to protect oneself from negative emotions such as cognitive dissonance (Westen et al., 2006). However, our prosociality factor included positive loadings for agreeableness, openness, extroversion, the factor of respect for others’ viewpoints from the comprehensive intellectual humility scale, and a negative loading for need for closure. Intellectual humility is associated with openness and agreeableness and prosocial emotions and behaviors like empathy and perspective-taking when encountering other opinions (Leary et al., 2017; Krumrei-Mancuso, 2017). It is likely that our prosociality factor has captured traits associated with the tendency to be more open and agreeable and care about other people, and that the positive association with emotional arousal reflects empathy, tolerance, and perspective-taking rather than dysregulation.

High curiosity factor scores also predicted less time spent reading credible sources, contrary to our predictions. This may indicate that more curious participants were motivated to take in more information more quickly, perhaps using a strategic exploratory behavior such as skimming for information that applies to a knowledge gap. People high in epistemic curiosity feel rewarded when they fill their knowledge gap, which may motivate them to seek more information to receive more reward. Since curiosity also boosts knowledge integration, this could be considered a more effective method of information acquisition. It is also likely that the curiosity factor is associated with other individual differences that correlate with faster reading, since it encompasses several facets related to positive experiences with science and knowledge, need for cognition, and interest in science, which are associated with greater access to education and cultural activities.

### Pro-science decision-making was predicted by topic engagement, information sharing, and education

**Fig. 10:**
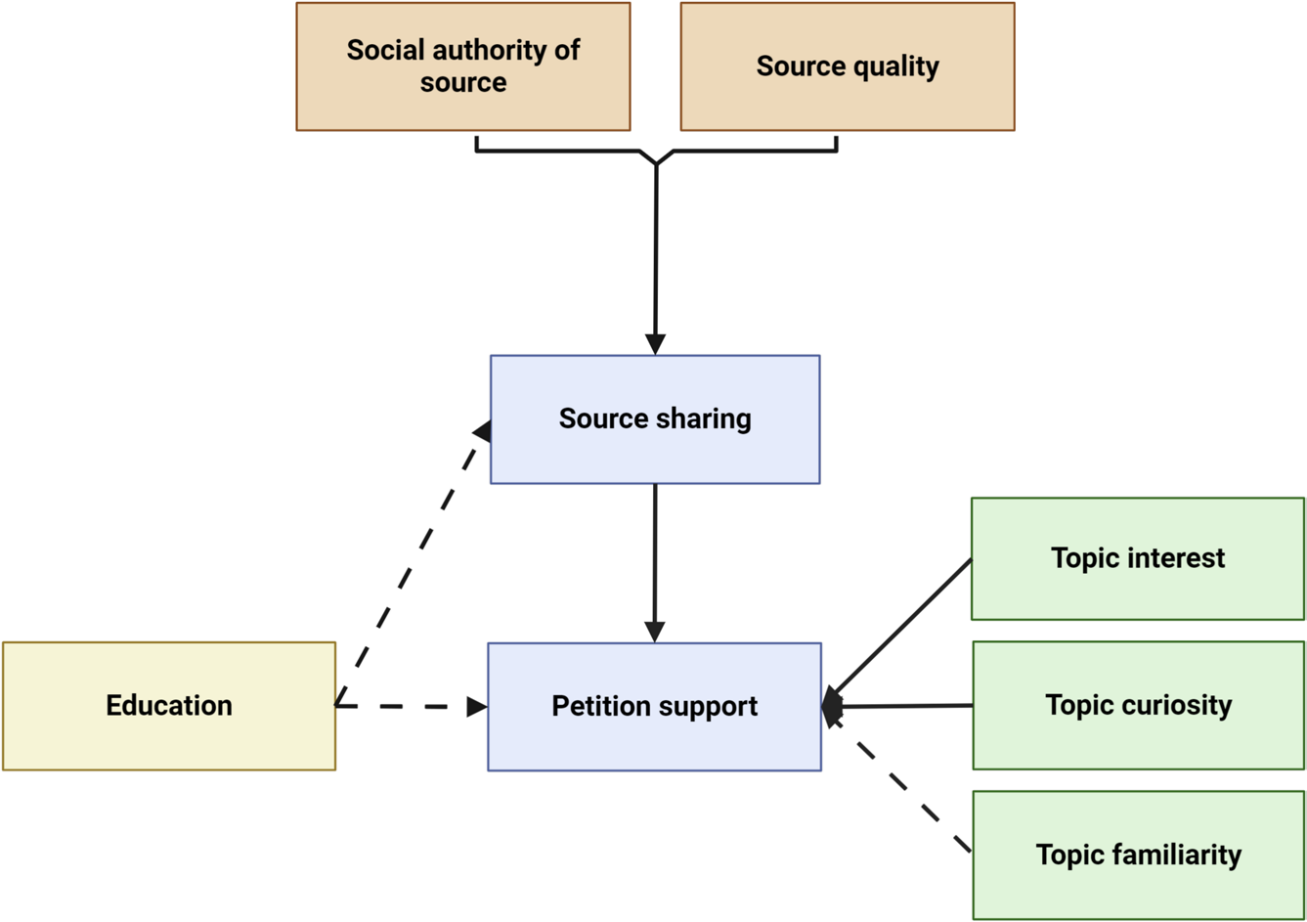
Conceptual diagram showing predictors of source sharing and petition support during the citizen’s initiative task. Solid lines represent positive effects; dashed lines represent negative effects. Brackets represent interactions. Colors indicate variable types: yellow are demographic variables; red are source features; green are topic related ratings; blue are ratings of willingness to share sources and support for the petition to reclassify peat as a renewable resource used as dependent variables. Created with Biorender.com

We found that while interest and curiosity about the topic of peat renewability predicted more support for the renewability petition, familiarity with the topic had the opposite effect, suggesting that interest and curiosity are at least partially independent from familiarity (Fig. 10). There is an important difference between the concepts of interest and engagement in science and technology, especially for adults whose science learning is generally informal and voluntary (Choung et al., 2020). “Engagement” can be behavioral, affective, or cognitive, and factors such as time and resources can interrupt a motivational push from interest to active engagement. We were interested in whether interest and curiosity would be associated with cognitive engagement (familiarity), which would in turn predict behavioral engagement (voting decision). Crucially, our familiarity item refers to the participant’s own subjective familiarity with the topic, not necessarily restricted to scientific or even correct information sources. Engagement with science and technology is partially predicted by epistemic beliefs; in particular, valuing knowledge and rational thinking (Choung et al., 2020). As we and others have demonstrated, cognitive dispositions such as need for cognition and actively open-minded thinking are also associated with motivation to engage with scientific information. Since our participants generally did not change their opinions through the course of the task, it is likely that most of their opinions were already formed before the task due to these individual differences in motivation to engage with either scientific or non-scientific sources on the topic.

Interestingly, petition support was positively associated with a willingness to share the articles with social networks. While we did not find the expected effect of prosociality on sharing behavior, people with less education were more likely to say they would share the articles, agreeing with previous work (Guess et al., 2019). This suggests that the ways information moves from person to person, especially online, are strongly driven by socio-cultural factors rather than the individual dispositions that influence how we engage with that information on an individual level. Additionally, the higher shareability of the higher quality sources suggests that in this task people intended to share good information, rather than being motivated by clickbait or their own bias. It is possible that the study’s task demands directed participants’ attention to finding good quality information in order to make an informed decision and/or because they were asked explicitly to rate the reliability of the evidence, as it has been demonstrated that instructing people to attend to information accuracy can encourage them to share better quality information (Pennycook & Rand, 2022).

However, there are other demographic and social factors which we did not measure; for example, participants with personal investment in the peat harvesting industry may be understandably more likely to support the petition despite other factors. These results underline the need to consider complex social-demographic backgrounds of participants that can affect their positions on science-related topics that have complex social impacts.

### Limitations

Because we selected real news articles for the citizen’s initiative task, we were not able to closely control the text features, and therefore text length was unbalanced between our categories of interest. It was a surprising and interesting finding that longer text length predicted higher ratings of convincingness, expertise and reliability. While many authors cite the potential for message length to be a cue for source reliability, few have quantified the effects through manipulation; rather, most simply match the texts for length. It is difficult to separate the effects of text length, number of arguments or claims, and complexity in real texts since scientific sources generally provide more information, contain more arguments, and can be more complex than non-scientific information sources.

While we have worked toward more ecological validity, the choice of topic and information sources was still controlled. Many other individual and contextual factors contribute to how people search for and evaluate scientific information in everyday life, such as being exposed to smaller pieces of information more frequently through news and social media, and the kinds of information that are served to them due to their prior interaction with media algorithms. The lack of change in petition support ratings that we found is in alignment with previous work showing that beliefs and opinions, once formed, are difficult to change (Stanley et al., 2018; Strømsø et al., 2017). However, the results also emphasize the importance of education and cognitive and emotional engagement with information. The body of literature on curiosity and epistemic emotions during learning suggests that promoting supportive, positive experiences with science may be key to cultivating more effective decision-making around scientific evidence.

Future work aiming to capture the nuances of naturalistic information-gathering and decision-making should consider the long timescale and constant, multimedia experience of modern information engagement when investigating effects over time. Other research designs might also prove useful for extending the ecological validity of future work, such as experience sampling and working with organizations in contexts where people already use information to make decisions, such as political or consumer polling, science museums, community events, or health centers.

### Implications for society and education

Curiosity and positive science attitudes, alongside cognitive control, emerged as important but surprising predictors of reading behavior; however, it is less clear how to interpret that behavior and how it relates to integration of new information and source evaluation. Corroborating previous work, we did not find a clear effect of cognitive skills on information seeking or source evaluation behavior.

We also identified a relationship between prosocial personality traits and respect for others’ viewpoints, which predicted emotional arousal during the information-seeking task. Being attuned to others’ perspectives could improve awareness of intentions and biases in shared information, and activation of empathy could lead to more effective interactions during disagreements.

We found that familiarity and education predicted a pro-science attitude in the final decision, again suggesting that engagement with information and access to scientific learning promotes scientific thinking. This is a positive sign for educational advocacy and initiatives to increase public access to science. This study also shows that social and demographic factors have strong effects on people’s science-related opinions and decision-making. Kaakinen et al. (2023) similarly found age and education level to be important predictors of science capital, which affects the ways a person can engage in a world where access to science and technology give privilege and power (Archer et al., 2015). Our finding that the social power of a source can bias people’s perception of its quality underscores the need for better scientific evidence evaluation skills for a society where science literacy is an important source of social privilege. Understanding the complex relationships between demographic factors, personal beliefs and motivations, individual differences in reasoning skills and styles, and engagement with science is critical for understanding how to facilitate access to high-quality scientific information for making evidence-based decisions on topics that impact people’s daily lives.

## Supplementary information

This article has supplementary material provided at the Open Science Framework (https://osf.io/72mut/).

## Data Availability

The data and materials are available at https://doi.org/10.17605/OSF.IO/72MUT and the study was preregistered in two phases at https://doi.org/10.17605/OSF.IO/G2MPE and https://doi.org/10.17605/OSF.IO/DTQAX.

## Appendix 1: Scale reliability

See Table 6.

**Table 6:** Cronbach’s alpha and McDonald’s omega for reliability of scales

Scale	Factor	$\alpha$	$\Omega$
Comprehensive intellectual humility scale	Independence of intellect and ego	0.87	0.93
	Openness to revising one’s viewpoint	0.85	0.87
	Respect for others’ viewpoints	0.84	0.90
	Lack of intellectual overconfidence	0.76	0.84
Science attitudes scale	Science identity	0.71	0.75
	Importance of science in everyday life	0.74	0.80
	Trust in science and scientists	0.58	0.62
Epistemic curiosity scale	Interest curiosity	0.80	0.88
	Deprivation curiosity	0.73	0.81
Science curiosity scale		0.62	0.77
Actively open-minded thinking scale		0.69	0.8
Need for cognition scale		0.88	0.91
Need for closure scale		0.84	0.87

Scales with internal factor structures are listed separately because they were used as separate constructs in the exploratory factor analysis

## Appendix 2: Correlation matrix for all variables

See Tables 7 and 8.

**Table 7:** Correlations between variables (Part 1)

Variable	Mean	SD	1	2	3	4	5	6	7	8	9	10	11
1. Agreeableness	4.86	1.12											
2. Conscientiousness (TIPI)	4.64	1.3	0.00										
3. Extraversion (TIPI)	4.29	1.48	0.14	0.16									
4. Emotional stability (TIPI)	4.46	1.6	0.21*	0.39***	0.03								
5. Openness (TIPI)	5.34	1.04	0.10	− 0.05	0.18*	− 0.09							
6. Interest curiosity (EC)	21.04	2.94	− 0.04	− 0.01	0.13	0.08	0.50***						
7. Deprivation curiosity (EC)	16.14	3.48	− 0.15	− 0.03	0.06	− 0.13	0.40***	0.60***					
8. Independence of intellect and ego (IH)	17.87	4.46	− 0.26**	0.13	0.01	0.21*	0.14	0.15	0.05				
9. Openness to revising one’s viewpoint (IH)	21.08	2.39	0.04	− 0.19*	0.00	− 0.16	0.30***	0.39***	0.34***	0.04			
10. Respect for others’ viewpoints (IH)	20.15	2.72	0.18*	0.11	0.20*	0.14	0.31***	0.30***	0.11	0.23**	0.19*		
11. Lack of intellectual overconfidence (IH)	17.9	2.83	0.14	− 0.19*	− 0.06	− 0.04	− 0.03	− 0.03	− 0.18*	− 0.11	0.24**	0.00	
12. Need for closure	51.32	10.52	0.00	0.10	− 0.28**	− 0.10	− 0.31***	− 0.28**	− 0.05	− 0.39***	− 0.07	− 0.31***	− 0.08
13. Need for cognition	81.38	12.04	− 0.10	0.13	0.26**	0.14	0.39***	0.63***	0.51***	0.27**	0.23**	0.20*	− 0.16
14. Actively open-minded thinking	42.17	4.16	− 0.21*	− 0.23**	− 0.17	− 0.04	0.13	0.33***	0.14	0.10	0.39***	− 0.04	0.18*
15. Randomness and probability	2.54	0.97	− 0.14	− 0.02	− 0.18*	− 0.06	− 0.13	0.15	0.10	− 0.04	0.28**	− 0.07	0.08

TIPI, Ten-item personality index; EC, Epistemic curiosity scale; IH, Comprehensive intellectual humility scale. Binary variables not included. *Indicates *p* < 0.05. **Indicates *p* < 0.01. ***Indicates *p* < 0.001

**Table 8:** Correlations between variables (Part 2)

Variable	12	13	14	15	16	17	18	19	20	21	22	23	24	25
1. Agreeableness														
2. Conscientiousness (TIPI)														
3. Extraversion (TIPI)														
4. Emotional stability (TIPI)														
5. Openness (TIPI)														
6. Interest curiosity (EC)														
7. Deprivation curiosity (EC)														
8. Independence of intellect and ego (IH)														
9. Openness to revising one’s viewpoint (IH)														
10. Respect for others’ viewpoints (IH)														
11. Lack of intellectual overconfidence (IH)														
12. Need for closure														
13. Need for cognition	− 0.41***													
14. Actively open-minded thinking	− 0.12	0.17												
15. Randomness and probability	0.07	0.10	0.27**											
16. Science curiosity	− 0.14	0.37***	0.17	0.21*										
17. Trust in science (SA)	0.06	0.22*	0.11	0.17	0.28**									
18. Importance of science (SA)	− 0.07	0.29***	0.28**	0.10	0.33***	0.33***								
19. Science identity (SA)	− 0.13	0.52***	0.10	0.29***	0.44***	0.21*	0.41***							
20. Fluid intelligence	− 0.07	0.09	0.16	0.33***	0.08	0.04	0.12	0.07						
21. Sensitivity *(d’)*	0.08	− 0.05	0.08	0.09	− 0.10	0.04	0.10	− 0.01	0.10					
22. Bias	− 0.15	0.01	− 0.03	− 0.10	0.05	− 0.06	− 0.03	0.04	− 0.19*	− 0.71***				
23. Global to local interference	− 0.01	0.08	− 0.01	0.05	0.04	− 0.01	0.09	0.18*	− 0.06	− 0.13	0.16			
24. Global to local precedence	0.05	− 0.11	− 0.06	− 0.20*	− 0.04	− 0.05	0.08	− 0.17	0.03	0.02	− 0.08	− 0.79***		
25. Perceptual switching rate	0.02	− 0.02	0.14	0.15	0.02	0.16	0.02	0.02	0.16	0.08	− 0.22*	− 0.06	0.00	
26. Flexibility	− 0.04	0.18*	0.10	0.05	0.08	0.06	0.17	0.12	0.02	0.19*	− 0.13	0.06	− 0.04	0.10

TIPI, Ten-item personality index; EC, Epistemic curiosity scale; IH, Comprehensive intellectual humility scale. Binary variables not included. *Indicates *p* < 0.05. **Indicates *p* < 0.01. ***Indicates *p* < 0.001

## Appendix 3: LMM tables

See Tables 9, 10, 11, 12, 13, 14, 15, and 16.

**Table 9:** Model for convincingness

Predictor	95% CI					
	Estimate	SE	LL	UL	*t*	*p*
*Fixed effects*						
(Intercept)	3.17	0.14	2.89	3.45	21.98	<0.001***
Personal	0.11	0.09	− 0.08	0.29	1.12	0.262
Non-credible	0.32	0.09	0.14	0.51	3.43	0.001***
Irrelevant	− 0.30	0.09	− 0.49	− 0.12	− 3.25	0.001***
Age	0.00	0.00	− 0.00	0.01	1.47	0.141
Lower university degree	− 0.17	0.11	− 0.39	0.04	− 1.56	0.120
Graduate degree	− 0.24	0.09	− 0.42	− 0.05	− 2.53	0.012*
Personal $\times$ Non-credible	− 1.51	0.13	− 1.77	− 1.25	− 11.35	<0.001***
Personal $\times$ Irrelevant	− 0.72	0.13	− 0.98	− 0.46	− 5.43	<0.001***
*Random effects*						
$\sigma ^2$	0.75					
$\tau 00$ Participant	0.12					
ICC	0.13					
N Participant	171					
Observations	1025					
Marginal $R^2$	0.245					
Conditional $R^2$	0.347					

*Indicates *p* < 0.05. ***Indicates *p* < 0.001

**Table 10:** Model for expertise

Predictor	95% CI					
	Estimate	SE	LL	UL	*t*	*p*
*Fixed effects*						
(Intercept)	2.77	0.15	2.47	3.07	18.01	<0.001***
Personal	0.25	0.09	0.08	0.43	2.81	0.005**
Non-credible	0.58	0.09	0.41	0.76	6.54	<0.001***
Irrelevant	0.01	0.09	− 0.16	0.19	0.13	0.896
Age	0.01	0.00	0.00	0.01	2.89	0.004**
Lower university degree	− 0.25	0.12	− 0.48	− 0.01	− 2.09	0.037*
Graduate degree	− 0.15	0.10	− 0.35	0.05	− 1.51	0.132
Personal $\times$ Non-credible	− 1.83	0.13	− 2.07	− 1.58	− 14.43	<0.001***
Personal $\times$ Irrelevant	− 1.32	0.13	− 1.57	− 1.07	− 10.45	<0.001***
*Random effects*						
$\sigma ^2$	0.68					
$\tau 00$ Participant	0.17					
ICC	0.20					
N Participant	171					
Observations	1025					
Marginal $R^2$	0.318					
Conditional $R^2$	0.455					

*Indicates *p* < 0.05. **Indicates *p* < 0.01. ***Indicates *p* < 0.001

**Table 11:** Model for reliability

Predictor	95% CI					
	Estimate	SE	LL	UL	*t*	*p*
*Fixed effects*						
(Intercept)	3.21	0.14	2.94	3.47	23.67	<0.001***
Personal	0.11	0.08	− 0.05	0.28	1.35	0.176
Non-credible	0.43	0.08	0.27	0.59	5.22	<0.001***
Irrelevant	− 0.02	0.08	− 0.18	0.14	− 0.27	0.791
Age	0.01	0.00	0.00	0.01	2.46	0.014**
Lower university degree	− 0.36	0.10	− 0.56	− 0.16	− 3.46	0.001***
Graduate degree	− 0.26	0.09	− 0.44	− 0.09	− 2.92	0.004**
Personal $\times$ Non-credible	− 1.39	0.12	− 1.62	− 1.16	− 11.83	<0.001***
Personal $\times$ Irrelevant	− 0.66	0.12	− 0.89	− 0.43	− 5.61	<0.001***
*Random effects*						
$\sigma ^2$	0.56					
$\tau 00$ Participant	0.12					
ICC	0.17					
N Participant	171					
Observations	991					
Marginal $R^2$	0.233					
Conditional $R^2$	0.367					

**Indicates *p* < 0.01. ***Indicates *p* < 0.001

**Table 12:** Model for reading rates

Predictor	Estimate	SE	t value	*p*
*Fixed effects*				
(Intercept)	6.19	0.17	36.14	<0.001***
Personal	0.45	0.094	4.85	<0.001***
Non-credible	0.16	0.092	1.69	0.091
Irrelevant	− 0.43	0.092	− 4.62	0.001***
Age	0.071	0.083	0.86	0.39
Lower university degree	− 0.40	0.23	− 1.70	0.089
Graduate degree	− 0.45	0.20	− 2.26	0.024*
Personal $\times$ Non-credible	0.19	0.13	1.38	0.17
Personal $\times$ Irrelevant	− 0.064	0.13	− 0.48	0.63
*Random effects*				
$\sigma ^2$	1.049			
$\tau 00$ Participant	1.065			
ICC	0.50			
N Participant	171			
Observations	1022			
Marginal $R^2$	0.081			
Conditional $R^2$	0.544			

*Indicates *p* < 0.05. **Indicates *p* < 0.01. ***Indicates *p* < 0.001

**Table 13:** Model for reading rates predicted by curiosity factor scores and source categories

Predictor	Estimate	SE	t	*p*
*Fixed effects*				
(Intercept)	5.67	0.17	32.51	<0.001***
Personal	0.25	0.10	2.52	0.012*
Non-credible	0.10	0.10	1.01	0.31
Irrelevant	− 0.47	0.10	− 4.68	<0.001***
Curiosity factor score	0.098	0.11	0.92	0.36
Age	0.07	0.079	0.88	0.38
Lower university degree	− 0.013	0.22	− 0.058	0.95
Graduate degree	− 0.002	0.2	− 0.01	0.99
Personal $\times$ Non-credible	0.42	0.15	2.88	0.0041**
Personal $\times$ Irrelevant	0.17	0.14	1.18	0.24
Personal $\times$ Curiosity	0.065	0.11	0.60	0.55
Non-credible $\times$ Curiosity	− 0.30	0.11	− 2.77	0.0056**
Irrelevant $\times$ Curiosity	− 0.083	0.11	− 0.77	0.44
Personal $\times$ Non-credible $\times$ Curiosity	− 0.0038	0.16	− 0.024	0.99
Personal $\times$ Irrelevant $\times$ Curiosity	− 0.026	0.15	− 0.17	0.86
*Random effects*				
$\sigma ^2$	1.05			
$\tau 00$ Participant	0.63			
ICC	0.38			
N Participant	126			
Observations	753			
Marginal $R^2$	0.086			
Conditional $R^2$	0.43			

*Indicates *p* < 0.05. **Indicates *p* < 0.01. ***Indicates *p* < 0.001

**Table 14:** Model for curiosity and source quality on reliability

Predictor	95% CI					
	Estimate	SE	LL	UL	*t*	*p*
*Fixed effects*						
(Intercept)	3.31	0.16	2.99	3.63	20.25	<0.001***
Personal	0.13	0.089	− 0.05	0.30	1.44	0.15
Non-credible	0.43	0.089	0.26	0.61	4.85	<0.001***
Irrelevant	− 0.52	0.089	− 0.23	0.12	− 0.58	0.56
Curiosity	− 0.0066	0.078	− 0.16	0.15	− 0.085	0.93
Age	0.0054	0.0028	− 0.00	0.01	1.89	0.061
Lower university degree	− 0.42	0.13	− 0.67	− 0.18	− 3.37	0.001***
Graduate degree	− 0.29	0.11	− 0.52	− 0.07	− 2.57	0.011*
Personal $\times$ Non-credible	− 1.51	0.13	− 1.75	− 1.26	− 11.87	<0.001***
Personal $\times$ Irrelevant	− 0.70	0.13	− 0.95	− 0.45	− 5.52	<0.001***
Personal $\times$ Curiosity	0.065	0.095	− 0.12	0.25	0.69	0.49
Non-credible $\times$ Curiosity	0.064	0.095	− 0.12	0.25	0.67	0.50
Irrelevant $\times$ Curiosity	− 0.05	0.095	− 0.24	0.14	− 0.53	0.60
Personal $\times$ Non-credible $\times$ Curiosity	− 0.19	0.13	− 0.45	0.08	− 1.39	0.17
Personal $\times$ Irrelevant $\times$ Curiosity	− 0.19	0.13	− 0.46	0.07	− 1.44	0.15
*Random effects*						
$\sigma ^2$	0.51					
$\tau 00$ Participant	0.13					
ICC	0.21					
N Participant	128					
Observations	762					
Marginal $R^2$	0.279					
Conditional $R^2$	0.430					

*Indicates *p* < 0.05. ***Indicates *p* < 0.001

**Table 15:** Model for prosociality on emotional arousal

Predictor	95% CI					
	Estimate	SE	LL	UL	*t*	*p*
*Fixed effects*						
(Intercept)	4.88	0.40	4.09	5.66	12.26	<0.001***
Personal	0.12	0.13	− 0.14	0.37	0.97	0.33
Non-credible	0.32	0.13	0.08	0.59	2.5	0.013*
Irrelevant	− 0.40	0.13	− 0.66	− 0.15	− 3.11	0.002**
Prosociality	0.43	0.17	0.07	0.74	2.59	0.01*
Age	0.019	0.0074	0.00	0.03	2.49	0.014*
Lower university degree	0.05	0.33	− 0.54	0.81	0.15	0.88
Graduate degree	− 0.12	0.30	− 0.63	0.58	− 0.39	0.69
Personal $\times$ Non-credible	− 0.91	0.18	− 1.28	− 0.56	− 5.044	<0.001***
Personal $\times$ Irrelevant	0.40	0.18	0.03	0.75	2.19	0.029*
Personal $\times$ Prosociality	− 0.072	0.15	− 0.38	0.23	− 0.47	0.64
Non-credible $\times$ Prosociality	− 0.13	0.15	− 0.44	0.17	− 0.83	0.41
Irrelevant $\times$ Prosociality	− 0.0064	0.15	− 0.30	0.31	− 0.042	0.97
Personal $\times$ Non-credible $\times$ Prosociality	0.13	0.22	− 0.28	0.58	0.62	0.54
Personal $\times$ Irrelevant $\times$ Prosociality	− 0.31	0.22	− 0.72	0.15	− 1.45	0.15
*Random effects*						
$\sigma ^2$	1.05					
$\tau 00$ Participant	1.41					
ICC	0.57					
N Participant	128					
Observations	768					
Marginal $R^2$	0.093					
Conditional $R^2$	0.61					

*Indicates *p* < 0.05. **Indicates *p* < 0.01. ***Indicates *p* < 0.001

**Table 16:** Model for sensitivity on expertise

Predictor	95% CI					
	Estimate	SE	LL	UL	*t*	*p*
*Fixed effects*						
(Intercept)	2.98	0.31	2.37	3.59	9.59	<0.001***
Personal	0.43	0.32	− 0.20	1.06	1.34	0.18
Non-credible	0.48	0.32	− 0.15	1.11	1.50	0.13
Irrelevant	− 0.58	0.32	− 1.21	0.05	− 1.80	0.072
Sensitivity *(d’)*	− 0.03	0.09	− 0.21	0.15	− 0.32	0.75
Age	0.01	0.00	0.00	0.01	2.05	0.040*
Lower university degree	− 0.29	0.14	− 0.57	− 0.02	− 2.08	0.038*
Graduate degree	− 0.13	0.13	− 0.38	0.12	− 1.04	0.297
Personal $\times$ Non-credible	− 2.29	0.45	− 3.18	− 1.40	− 5.06	<0.001***
Personal $\times$ Irrelevant	− 0.96	0.45	− 1.85	− 0.07	− 2.12	0.034*
Personal $\times$ sensitivity	− 0.06	0.12	− 0.29	0.17	− 0.55	0.59
Non-credible $\times$ sensitivity	0.03	0.12	− 0.19	0.26	0.29	0.77
Irrelevant $\times$ sensitivity	0.23	0.12	0.00	0.46	1.97	0.049*
Personal $\times$ Non-credible $\times$ sensitivity	0.14	0.17	− 0.19	0.46	0.84	0.40
Personal $\times$ Irrelevant $\times$ sensitivity	− 0.18	0.17	− 0.51	0.14	− 1.12	0.26
*Random effects*						
$\sigma ^2$	0.61					
$\tau 00$ Participant	0.17					
ICC	0.21					
N Participant	126					
Observations	756					
Marginal $R^2$	0.38					
Conditional $R^2$	0.51					

*Indicates *p* < 0.05. ***Indicates *p* < 0.001

## Appendix 4: Estimated marginal means and pairwise comparisons for source evaluation

See Tables 17 and 18.

**Table 17:** Estimated marginal means for convincingness, expertise and reliability ratings. Means for each factor level averaged over levels of education. Degrees-of-freedom method: Kenward-Roger

Source type	95% CI					
	Source quality	EMM	SE	df	Lower	Upper
*Convincingness*						
Authority	Credible	3.18	0.071	928	3.04	3.32
Authority	Non-credible	3.50	0.071	928	3.36	3.64
Authority	Irrelevant	2.88	0.071	928	2.74	3.02
Personal	Credible	3.29	0.071	928	3.15	3.43
Personal	Non-credible	2.10	0.071	929	1.96	2.24
Personal	Irrelevant	2.26	0.071	928	2.12	2.40
*Expertise*						
Authority	Credible	3.00	0.071	840	2.86	3.14
Authority	Non-credible	3.58	0.071	840	3.45	3.72
Authority	Irrelevant	3.01	0.071	840	2.87	3.15
Personal	Credible	3.25	0.071	840	3.11	3.39
Personal	Non-credible	2.01	0.071	840	1.87	2.15
Personal	Irrelevant	1.94	0.071	840	1.80	2.08
*Reliability*						
Authority	Credible	3.26	0.064	862	3.13	3.39
Authority	Non-credible	3.69	0.063	852	3.57	3.81
Authority	Irrelevant	3.24	0.064	855	3.11	3.36
Personal	Credible	3.37	0.064	861	3.25	3.50
Personal	Non-credible	2.42	0.065	870	2.29	2.54
Personal	Irrelevant	2.69	0.064	862	2.57	2.82

**Table 18:** Pairwise contrasts between levels of source type and source quality for convincingness, expertise and reliability ratings

Group	Contrast	Est	SE	df	*t*	*p*
Convincingness						
Credible	Authority–personal	− 0.11	0.09	849.01	− 1.12	0.83
Non-credible	Authority–personal	1.40	0.09	849.51	14.92	$$<0.001***$$
Irrelevant	Authority–personal	0.61	0.09	849.01	6.55	$$<0.001***$$
Authority	Credible–non-credible	− 0.32	0.09	849.01	− 3.43	0.01*
Authority	Credible–irrelevant	0.30	0.09	849.01	3.25	0.01*
Authority	Non-credible–irrelevant	0.63	0.09	849.01	6.68	$$<0.001***$$
Personal	Credible–non-credible	1.18	0.09	849.51	12.61	$<0.001$***
Personal	Credible–irrelevant	1.02	0.09	849.01	10.92	$<0.001$***
Personal	Non-credible–irrelevant	− 0.16	0.09	849.51	− 1.71	0.45
Expertise						
Credible	Authority–personal	− 0.25	0.09	849.01	− 2.81	0.04*
Non-credible	Authority–personal	1.57	0.09	849.41	17.58	$$<0.001***$$
Irrelevant	Authority–personal	1.07	0.09	849.01	11.97	$$<0.001***$$
Authority	Credible–non-credible	− 0.58	0.09	849.01	− 6.54	$$<0.001***$$
Authority	Credible–irrelevant	− 0.01	0.09	849.01	− 0.13	1.00
Authority	Non-credible–irrelevant	0.57	0.09	849.01	6.41	$$<0.001***$$
Personal	Credible–non-credible	1.24	0.09	849.41	13.86	$$<0.001***$$
Personal	Credible–irrelevant	1.31	0.09	849.01	14.65	$$<0.001***$$
Personal	Non-credible–irrelevant	0.07	0.09	849.41	0.76	0.96
Reliability						
Credible	Authority–personal	− 0.11	0.08	820.94	− 1.35	0.69
Non-credible	Authority–personal	1.28	0.08	820.98	15.39	$$<0.001***$$
Irrelevant	Authority–personal	0.54	0.08	817.96	6.59	$$<0.001***$$
Authority	Credible–non-credible	− 0.43	0.08	820.78	− 5.22	$$<0.001***$$
Authority	Credible–irrelevant	0.02	0.08	819.30	0.26	1.00
Authority	Non-credible–irrelevant	0.45	0.08	818.58	5.53	$$<0.001***$$
Personal	Credible–non-credible	0.96	0.08	823.57	11.46	$$<0.001***$$
Personal	Credible–irrelevant	0.68	0.08	820.65	8.17	$$<0.001***$$
Personal	Non-credible–irrelevant	− 0.28	0.08	822.71	− 3.34	0.01*

*Indicates *p* < 0.05. ***Indicates *p* < 0.001

## Appendix 5: Investigation of Effect of text length on ratings

Because we chose to use real articles in the citizen’s initiative task, we were not able to manipulate the text length. In the models for reading behavior, we aimed to control for text length by using words per minute as a measure of reading rates because it was likely that reading times and text lengths would be correlated. However, we did not expect text lengths to have an effect on the ratings of convincingness, expertise and reliability, and therefore we did not balance the text lengths within the source categories. The personal credible and authority non-credible sources were the longest, with 1146 and 892 words, respectively, while the other four sources were notably shorter, ranging from 327 to 422 words.

During the initial analysis, we noticed that the pattern of ratings was similar to the pattern of text lengths. Incorporating text length directly into these models to account for its interacting effects with source type and source quality was not possible due to the rank deficiency of the models. To investigate this effect, we fit separate models with only text length, age, and education as fixed effects and a random intercept was included at the participant level, confirming that text length was associated with higher ratings for convincingness, expertise, and reliability.

In order to understand the confounding effects of text length given this constraint, we conducted a series of model comparisons, using different techniques to answer the following questions:

### Does text length alone explain the observed effects on convincingness, expertise and reliability?

We investigate this by finding the proportions of variance explained by the model including only text length as a fixed effect of interest and the original model including source type and source quality as effects of interest. Conditional $R^2$ showed more variance explained by the original model including source type and quality (model 1) than the model including only text length (model 2), for all three rating types (Table 19).

**Table 19:** Variance explained by models using Nakagawa’s $R^2$ for mixed models

	Conditional $R^2$		
	Convincingness	Expertise	Reliability
Model 1	0.347	0.455	0.367
Model 2	0.191	0.256	0.232

### Do source type and source quality individually improve upon the minimal model including only text length, tested using the likelihood ratio test for nested models?

We computed likelihood ratio tests with sets of nested models comparing models with only text length versus models including both text length and source type, and text length and source quality, respectively. All comparisons showed the models with source categories improved fit over the models with only text length. For convincingness, the model with type and length ($\chi ^2$(2)=171.17, *p*< 0.001) and quality and length ($\chi ^2$(4)=183.94, *p*< 0.001). For expertise, the model with type and length ($\chi ^2$(2)=267.97, *p*< 0.001) and quality and length ($\chi ^2$(4)=268.37, *p*< 0.001). For reliability, the model with type and length ($\chi ^2$(2)=148.49, p< 0.001) and quality and length ($\chi ^2$(4)=161.64, *p*< 0.001).

### Which of these four candidate models minimizes the AIC?

Our original model specification including source type and source quality or the model with source quality and text length minimized AIC for convincingness and reliability (Table 20). For expertise, the model with text length and source type had a slightly lower AIC.

**Table 20:** AIC for candidate models

Model	AIC		
	Convincingness	Expertise	Reliability
Text length only	2916.18	2949.55	2547.72
Text length*source type	2749.01	2685.58	2403.23
Text length*source quality	2740.24	2689.18	2394.08
Source type*source quality	2740.24	2689.18	2394.08

Although text length likely has a genuine effect that slightly boosts the ratings for the two longer texts, our further analysis shows that the inclusion of source type and quality terms improve model fit. Models including those terms along with text length explain a greater proportion of variance than a model with only text length, suggesting that there are also important effects of source type and quality. In the interpretation of the main results, we consider where this uncontrolled “weighting” might inflate ratings. Importantly, text length did not explain much variance on its own, so we have evidence to support the main claims that source type and quality are important predictors for the ratings. In the absence of a solution to include text length in the full models, our original model structure suits the current data best; therefore, we report the original planned analyses and discuss where text length might influence interpretation.

## Abbreviations

AOT: Actively open-minded thinking
EC: Epistemic curiosity
EFA: Exploratory factor analysis
H-E: Equiprobability heuristic
H-R: Representativeness heuristic
IH: Intellectual humility
SA: Science attitudes
SAM: Self-assessment manikin
SD: Standard deviation
TIPI: Ten item personality index
UUT: Unusual uses test
WPM: Words per minute
